# Supplementary material for: Noradrenergic-dependent functions are associated with age-related locus coeruleus signal intensity differences
Source: Nat Commun. 2020 Apr 6;11:1712. doi: 10.1038/s41467-020-15410-w (PMC7136271; doi:10.1038/s41467-020-15410-w)
Supplement: Supplementary file 2 — Reporting Summary [file 41467_2020_15410_MOESM2_ESM.pdf]

natureresearch

Corresponding author(s): Kathy Y. Liu

Last updated by author(s): Feb 18, 2020

Reporting Summary

Nature Research wishes to improve the reproducibility of the work that we publish. This form provides structure for consistency and transparency in reporting. For further information on Nature Research policies, seeAuthors & Referees and theEditorial Policy Checklist .

Statistics

For all statistical analyses, confirm that the following items are present in the figure legend, table legend, main text, or Methods section.

n/a	Confirmed	
☐	☒	The exact sample size (*n*) for each experimental group/condition, given as a discrete number and unit of measurement
☐	☒	A statement on whether measurements were taken from distinct samples or whether the same sample was measured repeatedly
☐	☒	The statistical test(s) used AND whether they are one- or two-sided<br>*Only common tests should be described solely by name; describe more complex techniques in the Methods section.*
☐	☒	A description of all covariates tested
☐	☒	A description of any assumptions or corrections, such as tests of normality and adjustment for multiple comparisons
☐	☒	A full description of the statistical parameters including central tendency (e.g. means) or other basic estimates (e.g. regression coefficient) AND variation (e.g. standard deviation) or associated estimates of uncertainty (e.g. confidence intervals)
☐	☒	For null hypothesis testing, the test statistic (e.g. *F*, *t*, *r*) with confidence intervals, effect sizes, degrees of freedom and *P* value noted<br>*Give P values as exact values whenever suitable.*
☐	☒	For Bayesian analysis, information on the choice of priors and Markov chain Monte Carlo settings
☐	☒	For hierarchical and complex designs, identification of the appropriate level for tests and full reporting of outcomes
☐	☒	Estimates of effect sizes (e.g. Cohen's *d*, Pearson's *r*), indicating how they were calculated

Our web collection on statistics for biologists contains articles on many of the points above.

Software and code

Policy information about availability of computer code

Data collection	The data were obtained from Cam-CAN, an open-access dataset available at http://www.mrc-cbu.cam.ac.uk/datasets/camcan/. The LC signal intensity measures were obtained from MT-weighted as described in a previous study, which used ANTS v1.2 software for MRI image preprocessing.
Data analysis	The data were analyzed using R Studio 3.5.1 and lavaan package 0.6-3. The R code that supports the analyses within this paper are available at https://github.com/k-y-liu/SEM-CamCAN-LC-code.

For manuscripts utilizing custom algorithms or software that are central to the research but not yet described in published literature, software must be made available to editors/reviewers. We strongly encourage code deposition in a community repository (e.g. GitHub). See the Nature Research guidelines for submitting code & software for further information.

Data

Policy information about availability of data

All manuscripts must include a data availability statement. This statement should provide the following information, where applicable:

- Accession codes, unique identifiers, or web links for publicly available datasets
- A list of figures that have associated raw data
- A description of any restrictions on data availability

The Cam-CAN is an open-access dataset available at http://www.mrc-cbu.cam.ac.uk/datasets/camcan/. The Cam-CAN response inhibition (SSRT) data are not yet publicly available.The R code that supports the analyses within this paper are available at https://github.com/k-y-liu/SEM-CamCAN-LC-code.

Field-specific reporting

Please select the one below that is the best fit for your research. If you are not sure, read the appropriate sections before making your selection.

- [ ] Life sciences
- [x] Behavioural & social sciences
- [ ] Ecological, evolutionary & environmental sciences

For a reference copy of the document with all sections, see nature.com/documents/nr-reporting-summary-flat.pdf

Behavioural & social sciences study design

All studies must disclose on these points even when the disclosure is negative.

Study description	Cross-sectional quantitative analysis of lifespan data.
Research sample	The Cam-CAN dataset http://www.mrc-cbu.cam.ac.uk/datasets/camcan/ consisted of cognitively healthy individuals aged between 18-88years who were representative of the population.
Sampling strategy	The Cam-CAN data were drawn from the general population via Primary Care Trust (PCT)'s lists within the Cambridge City (UK) area.
Data collection	Data collection was completed prior to the study hypothesis or analysis plan being completed. The data collection procedures were a combination of MRI imaging, computerized and non-computerized (including pen and paper) assessments. A researcher was present during the testing.
Timing	The Cam-CAN launched in October 2010 and consisted of 3 stages. The second stage recruited n=700 (100 individuals from each decile) between January 2011 and April 2014, and 280 of these individuals (40 from each decile) completed stage 3 between August 2013 and October 2014.
Data exclusions	A full list of pre-established exclusion criteria for Cam-CAN can be found here doi: 10.1186/s12883-014-0204-1.We used data from 605 participants who had MT-weighted MRI imaging from a previous study https://doi.org/10.1016/j.neurobiolaging.2018.10.014 (18 individuals who had significant motion artifacts had already been excluded).
Non-participation	Apart from the excluded data described, no additional dropouts occurred.
Randomization	This was a cross-sectional analysis with no randomized allocation. The analysis included a multi-group analysis after dividing the sample into older and younger adults.

Reporting for specific materials, systems and methods

We require information from authors about some types of materials, experimental systems and methods used in many studies. Here, indicate whether each material, system or method listed is relevant to your study. If you are not sure if a list item applies to your research, read the appropriate section before selecting a response.

Materials & experimental systems

n/a	Involved in the study	
x		Antibodies
x		Eukaryotic cell lines
x		Palaeontology
x		Animals and other organisms
	x	Human research participants
x		Clinical data

Methods

n/a	Involved in the study	
x		ChIP-seq
x		Flow cytometry
	x	MRI-based neuroimaging

Human research participants

Policy information about studies involving human research participants

Population characteristics	See above.
Recruitment	The Cam-CAN data were drawn from the general population via Primary Care Trust (PCT)'s lists within the Cambridge City (UK) area. Participants whose Primary Care Physician felt were inappropriate to include did not take part in the study., which may have introduced some selection bias.
Ethics oversight	Ethical approval for the study was obtained from the Cambridgeshire 2 (now East of England - Cambridge Central) Research Ethics Committee (reference: 10/H0308/50), and all participants provided written informed consent prior to the study.

Note that full information on the approval of the study protocol must also be provided in the manuscript.

Magnetic resonance imaging

Experimental design

Design type	Resting state, MT-weighted and T1-weighted structural MRI
Design specifications	No tasks were completed during scanning.
Behavioral performance measures	A number of variables pertaining to each task were obtained., described in Table 4 in the manuscript.

Acquisition

Imaging type(s)	Structural
Field strength	3T
Sequence & imaging parameters	The MT-prepared spoiled gradient echo sequence with either repetition time (TR) = 30 ms or TR =50 ms (the TR=50 milliseconds sequences were used when the participant's SAR estimation for the TR=30 milliseconds sequences exceeded the stimulation limits); echo time = 5 milliseconds; flip angle =12; field of view =192 192 mm; voxel size = 1.5 mm isotropic; bandwidth =190 Hz/px; acquisition time of 2 minutes and 36 seconds per sequence for TR = 30 milliseconds, and 4 minutes and 19 seconds per sequence for TR =50 milliseconds. For MT weighting, a Gaussian shaped RF pulse with an offset frequency of 1950 Hz (bandwidth = 375 Hz, 500 flip angle, duration = 9984 microseconds) was used. A 3D T1-weighted structural image was also acquired for each participant using a magnetization-prepared rapid gradient echo sequence with the following parameters: TR = 2250 milliseconds, echo time =2.00 milliseconds, inversion time = 900 milliseconds; flip angle = 9; field of view = 256 240 192 mm; voxel size = 1 mm isotropic; generalized autoc alibrating partial parallel acquisition acceleration factor = 2; acquisition time of 4 minutes 32 seconds.
Area of acquisition	Whole brain scan

Diffusion MRI ☐ Used ☒ Not used

Preprocessing

Preprocessing software	MT-weighted images were upsampled to 0.8-mm isotropic resolution. MT-weighted images were then bias corrected (N4-ITK) using ANTS v.2.1.
Normalization	The bias corrected MT images were spatially normalized to a studywise space using a highly iterative coregistration routine available from the ANTS v2.1 software package (http://stnava. github.io/ANTs).
Normalization template	Group studywise space
Noise and artifact removal	Individual MT- and T1- weighted scans were visually inspected for movement artifacts independently by two authors
Volume censoring	Not performed for structural data.

Statistical modeling & inference

Model type and settings	Structural equation modelling, multigroup analysis.
Effect(s) tested	The models tested the relationship between multiple observed variables and the construct(s) of interest (latent variable (s)). The observed variables are combined to form a composite measure, with the assumption that this will be a more reliable estimate of the construct than any one item on its own. The study tested the hypothesis that putatively noradrenergic (NA)-dependent functions would be more strongly associated with LC signal intensity in older versus younger adults.

Specify type of analysis: ☐ Whole brain ☒ ROI-based ☐ Both

Anatomical location(s)	The LC ROI was determined as described in a previous study https://doi.org/10.1016/j.neurobiolaging.2018.10.014
Statistic type for inference (See Eklund et al. 2016)	Not performed.
Correction	Not performed.

Models & analysis

n/a	Involved in the study
☒	☐ Functional and/or effective connectivity
☒	☐ Graph analysis
☐	☒ Multivariate modeling or predictive analysis

Multivariate modeling and predictive analysis	We used structural equation modelling (SEM) to compare a second order, multidimensional model (where LC CR is an independent, observed variable connecting to two latent NA-dependent and NA-independent factors) to a unidimensional model (where LC CR relates to a single latent variable representing all cognitive and behavioral measures). We predicted that a two-factor model would provide the best fit for the data and that older adults would show a stronger relationship between LC CR and the NA-dependent compared to the NA-independent factor.
