# Supplementary material for: Noradrenergic-dependent functions are associated with age-related locus coeruleus signal intensity differences
Source: Nat Commun. 2020 Apr 6;11:1712. doi: 10.1038/s41467-020-15410-w (PMC7136271; doi:10.1038/s41467-020-15410-w)
Supplement: Supplementary file 3 — Source Data [file 41467_2020_15410_MOESM3_ESM.zip › Source data Table 1.rtf]

> anova(two.factor.model.fit, one.factor.fit)Scaled Chi Square Difference Test (method = "satorra.bentler.2001")                     Df    AIC    BIC  Chisq Chisq diff Df diff Pr(>Chisq)two.factor.model.fit 88 -14366 -14168 237.01                              one.factor.fit       90 -14366 -14177 241.42     4.0102       2     0.1346> summary (two.factor.model.fit, fit.measures=TRUE, standardized=TRUE, rsquare=TRUE)lavaan 0.6-3 ended normally after 193 iterations  Optimization method                           NLMINB  Number of free parameters                         45  Number of observations                           605  Number of missing patterns                        36  Estimator                                         ML      Robust  Model Fit Test Statistic                     237.012     237.135  Degrees of freedom                                88          88  P-value (Chi-square)                           0.000       0.000  Scaling correction factor                                  0.999    for the Yuan-Bentler correction (Mplus variant)User model versus baseline model:  Comparative Fit Index (CFI)                       NA          NA  Tucker-Lewis Index (TLI)                          NA          NA  Robust Comparative Fit Index (CFI)                            NA  Robust Tucker-Lewis Index (TLI)                               NALoglikelihood and Information Criteria:  Loglikelihood user model (H0)               7228.251    7228.251  Scaling correction factor                                  1.300    for the MLR correction  Loglikelihood unrestricted model (H1)       7346.757    7346.757  Scaling correction factor                                  1.101    for the MLR correction  Number of free parameters                         45          45  Akaike (AIC)                              -14366.503  -14366.503  Bayesian (BIC)                            -14168.268  -14168.268  Sample-size adjusted Bayesian (BIC)       -14311.132  -14311.132Root Mean Square Error of Approximation:  RMSEA                                          0.053       0.053  90 Percent Confidence Interval          0.045  0.061       0.045  0.061  P-value RMSEA <= 0.05                          0.268       0.266  Robust RMSEA                                               0.053  90 Percent Confidence Interval                             0.045  0.061Standardized Root Mean Square Residual:  SRMR                                           0.076       0.076Parameter Estimates:  Information                                 Observed  Observed information based on                Hessian  Standard Errors                   Robust.huber.whiteLatent Variables:                   Estimate  Std.Err  z-value  P(>|z|)   Std.lv  Std.all  NA_dep =~                                                                 EMoneg            1.000                               0.140    0.824    EMvneg            1.353    0.105   12.843    0.000    0.190    0.852    EMpneg            0.159    0.048    3.300    0.001    0.022    0.200    ERneg             0.510    0.115    4.445    0.000    0.072    0.353    ERnegreac         0.042    0.011    3.627    0.000    0.006    0.296    SSRTb            -0.020    0.042   -0.466    0.641   -0.003   -0.061    Hoteltask_Time   -0.468    0.063   -7.427    0.000   -0.066   -0.381    PSQI_score       -0.048    0.016   -2.981    0.003   -0.007   -0.179    education_age     0.121    0.015    8.239    0.000    0.017    0.427    Occ_score        -0.145    0.071   -2.051    0.040   -0.020   -0.129    STW_total         0.061    0.023    2.641    0.008    0.009    0.159  NA_indep =~                                                               Cattell.ttlscr    1.000                               0.051    0.776    semmbVsmnmb_pN   -0.163    0.106   -1.540    0.123   -0.008   -0.079    Faces_FAMnam      0.517    0.058    8.853    0.000    0.027    0.463Regressions:                   Estimate  Std.Err  z-value  P(>|z|)   Std.lv  Std.all  NA_dep ~                                                                  meRLC            -0.608    0.272   -2.236    0.025   -4.332   -0.120  NA_indep ~                                                                meRLC            -0.325    0.096   -3.384    0.001   -6.314   -0.175Covariances:                   Estimate  Std.Err  z-value  P(>|z|)   Std.lv  Std.all .NA_dep ~~                                                                .NA_indep          0.006    0.001    9.399    0.000    0.921    0.921Intercepts:                   Estimate  Std.Err  z-value  P(>|z|)   Std.lv  Std.all   .EMoneg            0.818    0.028   29.563    0.000    0.818    4.802   .EMvneg            0.611    0.039   15.800    0.000    0.611    2.744   .EMpneg            0.165    0.008   20.065    0.000    0.165    1.477   .ERneg             0.530    0.018   29.739    0.000    0.530    2.611   .ERnegreac         0.028    0.002   15.745    0.000    0.028    1.399   .SSRTb             0.172    0.005   38.156    0.000    0.172    3.833   .Hoteltask_Time    0.275    0.014   19.129    0.000    0.275    1.594   .PSQI_score        0.051    0.002   27.124    0.000    0.051    1.369   .education_age     0.213    0.003   62.333    0.000    0.213    5.342   .Occ_score         0.206    0.007   27.779    0.000    0.206    1.307   .STW_total         0.540    0.003  214.885    0.000    0.540   10.067   .Cattell.ttlscr    0.351    0.010   36.833    0.000    0.351    5.294   .semmbVsmnmb_pN    0.181    0.005   34.313    0.000    0.181    1.697   .Faces_FAMnam      0.251    0.005   45.783    0.000    0.251    4.379   .NA_dep            0.000                               0.000    0.000   .NA_indep          0.000                               0.000    0.000Variances:                   Estimate  Std.Err  z-value  P(>|z|)   Std.lv  Std.all   .EMoneg            0.009    0.001    7.331    0.000    0.009    0.321   .EMvneg            0.014    0.002    6.485    0.000    0.014    0.274   .EMpneg            0.012    0.001   13.571    0.000    0.012    0.960   .ERneg             0.036    0.005    7.553    0.000    0.036    0.876   .ERnegreac         0.000    0.000   10.739    0.000    0.000    0.913   .SSRTb             0.002    0.000    8.972    0.000    0.002    0.996   .Hoteltask_Time    0.025    0.002   14.117    0.000    0.025    0.855   .PSQI_score        0.001    0.000   12.125    0.000    0.001    0.968   .education_age     0.001    0.000    6.987    0.000    0.001    0.818   .Occ_score         0.024    0.002   10.506    0.000    0.024    0.983   .STW_total         0.003    0.000    9.350    0.000    0.003    0.975   .Cattell.ttlscr    0.002    0.000    6.333    0.000    0.002    0.397   .semmbVsmnmb_pN    0.011    0.001   13.648    0.000    0.011    0.994   .Faces_FAMnam      0.003    0.000   13.728    0.000    0.003    0.786   .NA_dep            0.019    0.003    7.029    0.000    0.986    0.986   .NA_indep          0.003    0.000    7.312    0.000    0.970    0.970R-Square:                   Estimate    EMoneg            0.679    EMvneg            0.726    EMpneg            0.040    ERneg             0.124    ERnegreac         0.087    SSRTb             0.004    Hoteltask_Time    0.145    PSQI_score        0.032    education_age     0.182    Occ_score         0.017    STW_total         0.025    Cattell.ttlscr    0.603    semmbVsmnmb_pN    0.006    Faces_FAMnam      0.214    NA_dep            0.014    NA_indep          0.030> summary(one.factor.fit,fit.measures=TRUE, standardized=TRUE, rsquare=TRUE, ci=TRUE)lavaan 0.6-3 ended normally after 176 iterations  Optimization method                           NLMINB  Number of free parameters                         43  Number of observations                           605  Number of missing patterns                        36  Estimator                                         ML      Robust  Model Fit Test Statistic                     241.422     241.011  Degrees of freedom                                90          90  P-value (Chi-square)                           0.000       0.000  Scaling correction factor                                  1.002    for the Yuan-Bentler correction (Mplus variant)User model versus baseline model:  Comparative Fit Index (CFI)                       NA          NA  Tucker-Lewis Index (TLI)                          NA          NA  Robust Comparative Fit Index (CFI)                            NA  Robust Tucker-Lewis Index (TLI)                               NALoglikelihood and Information Criteria:  Loglikelihood user model (H0)               7226.047    7226.047  Scaling correction factor                                  1.309    for the MLR correction  Loglikelihood unrestricted model (H1)       7346.757    7346.757  Scaling correction factor                                  1.101    for the MLR correction  Number of free parameters                         43          43  Akaike (AIC)                              -14366.093  -14366.093  Bayesian (BIC)                            -14176.669  -14176.669  Sample-size adjusted Bayesian (BIC)       -14313.183  -14313.183Root Mean Square Error of Approximation:  RMSEA                                          0.053       0.053  90 Percent Confidence Interval          0.045  0.061       0.045  0.061  P-value RMSEA <= 0.05                          0.277       0.282  Robust RMSEA                                               0.053  90 Percent Confidence Interval                             0.045  0.061Standardized Root Mean Square Residual:  SRMR                                           0.076       0.076Parameter Estimates:  Information                                 Observed  Observed information based on                Hessian  Standard Errors                   Robust.huber.whiteLatent Variables:                   Estimate  Std.Err  z-value  P(>|z|) ci.lower ci.upper   Std.lv  Std.all  factor =~                                                                                   EMoneg            1.000                               1.000    1.000    0.140    0.819    EMvneg            1.357    0.103   13.196    0.000    1.156    1.559    0.189    0.850    EMpneg            0.161    0.048    3.321    0.001    0.066    0.256    0.022    0.201    ERneg             0.499    0.111    4.489    0.000    0.281    0.717    0.070    0.343    ERnegreac         0.043    0.011    4.020    0.000    0.022    0.064    0.006    0.306    SSRTb            -0.023    0.041   -0.568    0.570   -0.104    0.057   -0.003   -0.072    Hoteltask_Time   -0.473    0.063   -7.529    0.000   -0.596   -0.350   -0.066   -0.382    PSQI_score       -0.046    0.016   -2.958    0.003   -0.077   -0.016   -0.006   -0.172    education_age     0.119    0.014    8.415    0.000    0.091    0.147    0.017    0.416    Occ_score        -0.135    0.068   -1.989    0.047   -0.269   -0.002   -0.019   -0.120    STW_total         0.057    0.022    2.588    0.010    0.014    0.101    0.008    0.149    Cattell.ttlscr    0.347    0.029   11.925    0.000    0.290    0.404    0.048    0.731    semmbVsmnmb_pN   -0.055    0.039   -1.417    0.157   -0.131    0.021   -0.008   -0.072    Faces_FAMnam      0.187    0.022    8.425    0.000    0.143    0.230    0.026    0.454Regressions:                   Estimate  Std.Err  z-value  P(>|z|) ci.lower ci.upper   Std.lv  Std.all  factor ~                                                                                    meRLC            -0.758    0.243   -3.111    0.002   -1.235   -0.280   -5.428   -0.150Intercepts:                   Estimate  Std.Err  z-value  P(>|z|) ci.lower ci.upper   Std.lv  Std.all   .EMoneg            0.832    0.025   33.044    0.000    0.783    0.881    0.832    4.884   .EMvneg            0.631    0.035   17.773    0.000    0.561    0.700    0.631    2.830   .EMpneg            0.167    0.008   20.031    0.000    0.151    0.183    0.167    1.498   .ERneg             0.536    0.017   32.197    0.000    0.503    0.568    0.536    2.643   .ERnegreac         0.028    0.002   16.523    0.000    0.025    0.032    0.028    1.434   .SSRTb             0.172    0.005   36.484    0.000    0.163    0.181    0.172    3.822   .Hoteltask_Time    0.268    0.014   19.759    0.000    0.241    0.295    0.268    1.554   .PSQI_score        0.051    0.002   27.369    0.000    0.047    0.055    0.051    1.354   .education_age     0.215    0.003   68.129    0.000    0.208    0.221    0.215    5.380   .Occ_score         0.205    0.007   27.315    0.000    0.190    0.219    0.205    1.299   .STW_total         0.541    0.002  216.646    0.000    0.536    0.546    0.541   10.079   .Cattell.ttlscr    0.345    0.009   39.408    0.000    0.328    0.362    0.345    5.208   .semmbVsmnmb_pN    0.182    0.005   36.150    0.000    0.172    0.192    0.182    1.707   .Faces_FAMnam      0.249    0.005   48.764    0.000    0.239    0.259    0.249    4.337   .factor            0.000                               0.000    0.000    0.000    0.000Variances:                   Estimate  Std.Err  z-value  P(>|z|) ci.lower ci.upper   Std.lv  Std.all   .EMoneg            0.010    0.001    7.382    0.000    0.007    0.012    0.010    0.329   .EMvneg            0.014    0.002    6.755    0.000    0.010    0.018    0.014    0.278   .EMpneg            0.012    0.001   13.551    0.000    0.010    0.014    0.012    0.960   .ERneg             0.036    0.005    7.618    0.000    0.027    0.046    0.036    0.882   .ERnegreac         0.000    0.000   10.815    0.000    0.000    0.000    0.000    0.906   .SSRTb             0.002    0.000    8.999    0.000    0.002    0.002    0.002    0.995   .Hoteltask_Time    0.025    0.002   14.143    0.000    0.022    0.029    0.025    0.854   .PSQI_score        0.001    0.000   12.078    0.000    0.001    0.002    0.001    0.970   .education_age     0.001    0.000    6.947    0.000    0.001    0.002    0.001    0.827   .Occ_score         0.024    0.002   10.525    0.000    0.020    0.029    0.024    0.986   .STW_total         0.003    0.000    9.381    0.000    0.002    0.003    0.003    0.978   .Cattell.ttlscr    0.002    0.000   10.378    0.000    0.002    0.002    0.002    0.465   .semmbVsmnmb_pN    0.011    0.001   13.684    0.000    0.010    0.013    0.011    0.995   .Faces_FAMnam      0.003    0.000   14.158    0.000    0.002    0.003    0.003    0.794   .factor            0.019    0.003    6.943    0.000    0.014    0.024    0.977    0.977R-Square:                   Estimate    EMoneg            0.671    EMvneg            0.722    EMpneg            0.040    ERneg             0.118    ERnegreac         0.094    SSRTb             0.005    Hoteltask_Time    0.146    PSQI_score        0.030    education_age     0.173    Occ_score         0.014    STW_total         0.022    Cattell.ttlscr    0.535    semmbVsmnmb_pN    0.005    Faces_FAMnam      0.206    factor            0.023
