# Supplementary material for: Noradrenergic-dependent functions are associated with age-related locus coeruleus signal intensity differences
Source: Nat Commun. 2020 Apr 6;11:1712. doi: 10.1038/s41467-020-15410-w (PMC7136271; doi:10.1038/s41467-020-15410-w)
Supplement: Supplementary file 3 — Source Data [file 41467_2020_15410_MOESM3_ESM.zip › Source Data Table 2.rtf]

*AGE-ADJUSTED MULTI-GROUP UNIDIMENSIONAL MODEL - WHOLE LC> unconstrained.model.uni<-'+ factor=~ EMoneg+EMvneg +EMpneg+ERneg + ERnegreac +SSRTb+ Hoteltask_Time+ PSQI_score+education_age+Occ_score+STW_total+Cattell.totalscore+semambVsemunamb_pNo + Faces_FAMnam + factor ~ c(LC_effect_young,LC_effect_old)*meRLC+ factor~age_years+ meRLC~age_years'> unconstrained.model.uni.fit<- cfa(unconstrained.model.uni, data=ourdata1,  group="agecat",group.equal = c("loadings"),missing = "ML", estimator='mlr')Warning messages:1: In lav_data_full(data = data, group = group, cluster = cluster,  :  lavaan WARNING: due to missing values, some pairwise combinations have less than 10% coverage2: In lav_data_full(data = data, group = group, cluster = cluster,  :  lavaan WARNING: due to missing values, some pairwise combinations have less than 10% coverage> summary(unconstrained.model.uni.fit,fit.measures=TRUE, standardized=TRUE, rsquare=TRUE, ci=TRUE)lavaan 0.6-3 ended normally after 369 iterations  Optimization method                           NLMINB  Number of free parameters                         94  Number of equality constraints                    13  Number of observations per group           older                                            269  younger                                          336  Number of missing patterns per group       older                                             25  younger                                           24  Estimator                                         ML      Robust  Model Fit Test Statistic                     619.820     635.984  Degrees of freedom                               219         219  P-value (Chi-square)                           0.000       0.000  Scaling correction factor                                  0.975    for the Yuan-Bentler correction (Mplus variant)Chi-square for each group:  older                                        265.642     272.570  younger                                      354.178     363.414User model versus baseline model:  Comparative Fit Index (CFI)                       NA          NA  Tucker-Lewis Index (TLI)                          NA          NA  Robust Comparative Fit Index (CFI)                            NA  Robust Tucker-Lewis Index (TLI)                               NALoglikelihood and Information Criteria:  Loglikelihood user model (H0)               8915.016    8915.016  Scaling correction factor                                  1.119    for the MLR correction  Loglikelihood unrestricted model (H1)       9224.927    9224.927  Scaling correction factor                                  1.084    for the MLR correction  Number of free parameters                         81          81  Akaike (AIC)                              -17668.033  -17668.033  Bayesian (BIC)                            -17311.209  -17311.209  Sample-size adjusted Bayesian (BIC)       -17568.364  -17568.364Root Mean Square Error of Approximation:  RMSEA                                          0.078       0.079  90 Percent Confidence Interval          0.071  0.085       0.072  0.087  P-value RMSEA <= 0.05                          0.000       0.000  Robust RMSEA                                               0.078  90 Percent Confidence Interval                             0.071  0.085Standardized Root Mean Square Residual:  SRMR                                           0.117       0.117Parameter Estimates:  Information                                 Observed  Observed information based on                Hessian  Standard Errors                   Robust.huber.whiteGroup 1 [older]:Latent Variables:                   Estimate  Std.Err  z-value  P(>|z|) ci.lower ci.upper   Std.lv  Std.all  factor =~                                                                                   EMoneg            1.000                               1.000    1.000    0.129    0.744    EMvneg  (.p2.)    1.255    0.133    9.422    0.000    0.994    1.516    0.161    0.789    EMpneg  (.p3.)    0.082    0.072    1.136    0.256   -0.059    0.223    0.010    0.088    ERneg   (.p4.)    0.462    0.191    2.417    0.016    0.087    0.836    0.059    0.268    ERnegrc (.p5.)    0.020    0.020    0.993    0.321   -0.020    0.060    0.003    0.129    SSRTb   (.p6.)    0.036    0.069    0.522    0.602   -0.099    0.172    0.005    0.096    Htlts_T (.p7.)   -0.466    0.099   -4.702    0.000   -0.661   -0.272   -0.060   -0.323    PSQI_sc (.p8.)   -0.056    0.025   -2.226    0.026   -0.106   -0.007   -0.007   -0.186    edctn_g (.p9.)    0.160    0.031    5.082    0.000    0.098    0.221    0.021    0.446    Occ_scr (.10.)   -0.233    0.118   -1.978    0.048   -0.464   -0.002   -0.030   -0.198    STW_ttl (.11.)    0.151    0.043    3.515    0.000    0.067    0.235    0.019    0.351    Cttll.t (.12.)    0.307    0.054    5.725    0.000    0.202    0.412    0.039    0.668    smmbV_N (.13.)   -0.036    0.060   -0.602    0.547   -0.154    0.081   -0.005   -0.041    Fcs_FAM (.14.)    0.112    0.034    3.295    0.001    0.045    0.179    0.014    0.249Regressions:                   Estimate  Std.Err  z-value  P(>|z|) ci.lower ci.upper   Std.lv  Std.all  factor ~                                                                                    meRLC   (LC__)    0.678    0.312    2.173    0.030    0.067    1.290    5.277    0.156    age_yrs          -0.804    0.148   -5.429    0.000   -1.094   -0.513   -6.253   -0.508  meRLC ~                                                                                     age_yrs          -0.032    0.021   -1.516    0.129   -0.073    0.009   -0.032   -0.087Intercepts:                   Estimate  Std.Err  z-value  P(>|z|) ci.lower ci.upper   Std.lv  Std.all   .EMoneg            1.166    0.115   10.145    0.000    0.941    1.391    1.166    6.748   .EMvneg            1.019    0.151    6.745    0.000    0.723    1.315    1.019    4.988   .EMpneg            0.175    0.041    4.276    0.000    0.095    0.255    0.175    1.467   .ERneg             0.689    0.125    5.513    0.000    0.444    0.934    0.689    3.107   .ERnegreac         0.029    0.012    2.370    0.018    0.005    0.053    0.029    1.441   .SSRTb             0.199    0.033    5.940    0.000    0.133    0.265    0.199    4.112   .Hoteltask_Time    0.111    0.068    1.627    0.104   -0.023    0.245    0.111    0.599   .PSQI_score        0.029    0.011    2.598    0.009    0.007    0.051    0.029    0.741   .education_age     0.278    0.016   16.851    0.000    0.246    0.310    0.278    6.040   .Occ_score         0.102    0.050    2.042    0.041    0.004    0.200    0.102    0.676   .STW_total         0.622    0.016   38.254    0.000    0.590    0.654    0.622   11.264   .Cattell.ttlscr    0.431    0.043    9.917    0.000    0.346    0.516    0.431    7.305   .semmbVsmnmb_pN    0.176    0.030    5.841    0.000    0.117    0.235    0.176    1.562   .Faces_FAMnam      0.265    0.024   11.131    0.000    0.219    0.312    0.265    4.595   .meRLC             0.122    0.015    8.194    0.000    0.093    0.151    0.122    4.120   .factor            0.000                               0.000    0.000    0.000    0.000Variances:                   Estimate  Std.Err  z-value  P(>|z|) ci.lower ci.upper   Std.lv  Std.all   .EMoneg            0.013    0.002    5.662    0.000    0.009    0.018    0.013    0.447   .EMvneg            0.016    0.003    5.623    0.000    0.010    0.021    0.016    0.378   .EMpneg            0.014    0.002    9.145    0.000    0.011    0.017    0.014    0.992   .ERneg             0.046    0.010    4.548    0.000    0.026    0.065    0.046    0.928   .ERnegreac         0.000    0.000    6.828    0.000    0.000    0.001    0.000    0.983   .SSRTb             0.002    0.000    5.933    0.000    0.002    0.003    0.002    0.991   .Hoteltask_Time    0.031    0.003    9.502    0.000    0.025    0.037    0.031    0.896   .PSQI_score        0.001    0.000    8.815    0.000    0.001    0.002    0.001    0.966   .education_age     0.002    0.000    4.565    0.000    0.001    0.002    0.002    0.801   .Occ_score         0.022    0.003    7.069    0.000    0.016    0.028    0.022    0.961   .STW_total         0.003    0.000    5.923    0.000    0.002    0.004    0.003    0.877   .Cattell.ttlscr    0.002    0.000    7.149    0.000    0.001    0.002    0.002    0.554   .semmbVsmnmb_pN    0.013    0.002    8.024    0.000    0.010    0.016    0.013    0.998   .Faces_FAMnam      0.003    0.000   11.675    0.000    0.003    0.004    0.003    0.938   .meRLC             0.001    0.000   10.119    0.000    0.001    0.001    0.001    0.992   .factor            0.012    0.003    3.839    0.000    0.006    0.018    0.704    0.704R-Square:                   Estimate    EMoneg            0.553    EMvneg            0.622    EMpneg            0.008    ERneg             0.072    ERnegreac         0.017    SSRTb             0.009    Hoteltask_Time    0.104    PSQI_score        0.034    education_age     0.199    Occ_score         0.039    STW_total         0.123    Cattell.ttlscr    0.446    semmbVsmnmb_pN    0.002    Faces_FAMnam      0.062    meRLC             0.008    factor            0.296Group 2 [younger]:Latent Variables:                   Estimate  Std.Err  z-value  P(>|z|) ci.lower ci.upper   Std.lv  Std.all  factor =~                                                                                   EMoneg            1.000                               1.000    1.000    0.079    0.663    EMvneg  (.p2.)    1.255    0.133    9.422    0.000    0.994    1.516    0.099    0.634    EMpneg  (.p3.)    0.082    0.072    1.136    0.256   -0.059    0.223    0.006    0.063    ERneg   (.p4.)    0.462    0.191    2.417    0.016    0.087    0.836    0.036    0.209    ERnegrc (.p5.)    0.020    0.020    0.993    0.321   -0.020    0.060    0.002    0.090    SSRTb   (.p6.)    0.036    0.069    0.522    0.602   -0.099    0.172    0.003    0.069    Htlts_T (.p7.)   -0.466    0.099   -4.702    0.000   -0.661   -0.272   -0.037   -0.243    PSQI_sc (.p8.)   -0.056    0.025   -2.226    0.026   -0.106   -0.007   -0.004   -0.122    edctn_g (.p9.)    0.160    0.031    5.082    0.000    0.098    0.221    0.013    0.389    Occ_scr (.10.)   -0.233    0.118   -1.978    0.048   -0.464   -0.002   -0.018   -0.113    STW_ttl (.11.)    0.151    0.043    3.515    0.000    0.067    0.235    0.012    0.230    Cttll.t (.12.)    0.307    0.054    5.725    0.000    0.202    0.412    0.024    0.532    smmbV_N (.13.)   -0.036    0.060   -0.602    0.547   -0.154    0.081   -0.003   -0.028    Fcs_FAM (.14.)    0.112    0.034    3.295    0.001    0.045    0.179    0.009    0.192Regressions:                   Estimate  Std.Err  z-value  P(>|z|) ci.lower ci.upper   Std.lv  Std.all  factor ~                                                                                    meRLC   (LC__)   -0.257    0.259   -0.995    0.320   -0.765    0.250   -3.277   -0.080    age_yrs          -0.197    0.084   -2.357    0.018   -0.361   -0.033   -2.509   -0.260  meRLC ~                                                                                     age_yrs           0.104    0.012    8.377    0.000    0.080    0.128    0.104    0.438Intercepts:                   Estimate  Std.Err  z-value  P(>|z|) ci.lower ci.upper   Std.lv  Std.all   .EMoneg            0.941    0.030   31.753    0.000    0.883    0.999    0.941    7.941   .EMvneg            0.778    0.041   19.080    0.000    0.698    0.858    0.778    5.005   .EMpneg            0.181    0.012   15.548    0.000    0.158    0.204    0.181    1.777   .ERneg             0.588    0.025   23.298    0.000    0.538    0.637    0.588    3.385   .ERnegreac         0.033    0.003   13.083    0.000    0.028    0.038    0.033    1.864   .SSRTb             0.171    0.009   20.112    0.000    0.154    0.188    0.171    4.139   .Hoteltask_Time    0.216    0.017   12.965    0.000    0.183    0.248    0.216    1.430   .PSQI_score        0.046    0.003   15.397    0.000    0.040    0.052    0.046    1.263   .education_age     0.230    0.005   48.978    0.000    0.221    0.239    0.230    7.129   .Occ_score         0.186    0.013   14.704    0.000    0.161    0.210    0.186    1.141   .STW_total         0.546    0.005  118.049    0.000    0.537    0.555    0.546   10.587   .Cattell.ttlscr    0.388    0.010   38.070    0.000    0.368    0.408    0.388    8.576   .semmbVsmnmb_pN    0.176    0.008   22.214    0.000    0.161    0.192    0.176    1.733   .Faces_FAMnam      0.270    0.005   58.182    0.000    0.261    0.279    0.270    5.874   .meRLC             0.046    0.005    9.447    0.000    0.036    0.055    0.046    1.860   .factor            0.000                               0.000    0.000    0.000    0.000Variances:                   Estimate  Std.Err  z-value  P(>|z|) ci.lower ci.upper   Std.lv  Std.all   .EMoneg            0.008    0.002    4.791    0.000    0.005    0.011    0.008    0.560   .EMvneg            0.014    0.003    4.941    0.000    0.009    0.020    0.014    0.598   .EMpneg            0.010    0.001   10.401    0.000    0.008    0.012    0.010    0.996   .ERneg             0.029    0.003    9.114    0.000    0.023    0.035    0.029    0.956   .ERnegreac         0.000    0.000    8.896    0.000    0.000    0.000    0.000    0.992   .SSRTb             0.002    0.000    7.176    0.000    0.001    0.002    0.002    0.995   .Hoteltask_Time    0.021    0.002   11.516    0.000    0.018    0.025    0.021    0.941   .PSQI_score        0.001    0.000    8.312    0.000    0.001    0.002    0.001    0.985   .education_age     0.001    0.000    8.644    0.000    0.001    0.001    0.001    0.849   .Occ_score         0.026    0.003    7.853    0.000    0.020    0.033    0.026    0.987   .STW_total         0.003    0.000    6.235    0.000    0.002    0.003    0.003    0.947   .Cattell.ttlscr    0.001    0.000    9.325    0.000    0.001    0.002    0.001    0.717   .semmbVsmnmb_pN    0.010    0.001   12.896    0.000    0.009    0.012    0.010    0.999   .Faces_FAMnam      0.002    0.000    8.301    0.000    0.002    0.003    0.002    0.963   .meRLC             0.000    0.000   10.401    0.000    0.000    0.001    0.000    0.809   .factor            0.006    0.002    2.932    0.003    0.002    0.009    0.908    0.908R-Square:                   Estimate    EMoneg            0.440    EMvneg            0.402    EMpneg            0.004    ERneg             0.044    ERnegreac         0.008    SSRTb             0.005    Hoteltask_Time    0.059    PSQI_score        0.015    education_age     0.151    Occ_score         0.013    STW_total         0.053    Cattell.ttlscr    0.283    semmbVsmnmb_pN    0.001    Faces_FAMnam      0.037    meRLC             0.191    factor            0.092*NON AGE-ADJUSTED MULTIGROUP UNIDIMENSIONAL MODEL - WHOLE LC> unconstrained.model.noage<-'+ factor=~ EMoneg+EMvneg +EMpneg+ERneg + ERnegreac +SSRTb+ Hoteltask_Time+ PSQI_score+education_age+Occ_score+STW_total+Cattell.totalscore+semambVsemunamb_pNo + Faces_FAMnam + factor ~ c(LC_effect_young,LC_effect_old)*meRLC'> unconstrained.noage.fit<- cfa(unconstrained.model.noage, data=ourdata1,  group="agecat",group.equal = c("loadings"),missing = "ML", estimator='mlr')Warning messages:1: In lav_data_full(data = data, group = group, cluster = cluster,  :  lavaan WARNING: due to missing values, some pairwise combinations have less than 10% coverage2: In lav_data_full(data = data, group = group, cluster = cluster,  :  lavaan WARNING: due to missing values, some pairwise combinations have less than 10% coverage> summary(unconstrained.noage.fit,fit.measures=TRUE, standardized=TRUE, rsquare=TRUE, ci=TRUE)lavaan 0.6-3 ended normally after 320 iterations  Optimization method                           NLMINB  Number of free parameters                         86  Number of equality constraints                    13  Number of observations per group           older                                            269  younger                                          336  Number of missing patterns per group       older                                             25  younger                                           24  Estimator                                         ML      Robust  Model Fit Test Statistic                     381.937     370.039  Degrees of freedom                               193         193  P-value (Chi-square)                           0.000       0.000  Scaling correction factor                                  1.032    for the Yuan-Bentler correction (Mplus variant)Chi-square for each group:  older                                        155.911     151.054  younger                                      226.027     218.985User model versus baseline model:  Comparative Fit Index (CFI)                       NA          NA  Tucker-Lewis Index (TLI)                          NA          NA  Robust Comparative Fit Index (CFI)                            NA  Robust Tucker-Lewis Index (TLI)                               NALoglikelihood and Information Criteria:  Loglikelihood user model (H0)               7519.795    7519.795  Scaling correction factor                                  1.125    for the MLR correction  Loglikelihood unrestricted model (H1)       7710.764    7710.764  Scaling correction factor                                  1.138    for the MLR correction  Number of free parameters                         73          73  Akaike (AIC)                              -14893.590  -14893.590  Bayesian (BIC)                            -14572.008  -14572.008  Sample-size adjusted Bayesian (BIC)       -14803.765  -14803.765Root Mean Square Error of Approximation:  RMSEA                                          0.057       0.055  90 Percent Confidence Interval          0.048  0.065       0.047  0.063  P-value RMSEA <= 0.05                          0.087       0.155  Robust RMSEA                                               0.056  90 Percent Confidence Interval                             0.047  0.065Standardized Root Mean Square Residual:  SRMR                                           0.116       0.116Parameter Estimates:  Information                                 Observed  Observed information based on                Hessian  Standard Errors                   Robust.huber.whiteGroup 1 [older]:Latent Variables:                   Estimate  Std.Err  z-value  P(>|z|) ci.lower ci.upper   Std.lv  Std.all  factor =~                                                                                   EMoneg            1.000                               1.000    1.000    0.126    0.740    EMvneg  (.p2.)    1.224    0.128    9.603    0.000    0.975    1.474    0.155    0.767    EMpneg  (.p3.)    0.066    0.075    0.878    0.380   -0.081    0.212    0.008    0.069    ERneg   (.p4.)    0.284    0.212    1.340    0.180   -0.131    0.699    0.036    0.160    ERnegrc (.p5.)   -0.009    0.019   -0.476    0.634   -0.046    0.028   -0.001   -0.056    SSRTb   (.p6.)    0.080    0.073    1.087    0.277   -0.064    0.223    0.010    0.204    Htlts_T (.p7.)   -0.449    0.102   -4.385    0.000   -0.649   -0.248   -0.057   -0.304    PSQI_sc (.p8.)   -0.066    0.026   -2.488    0.013   -0.117   -0.014   -0.008   -0.212    edctn_g (.p9.)    0.185    0.034    5.428    0.000    0.118    0.252    0.023    0.513    Occ_scr (.10.)   -0.356    0.118   -3.017    0.003   -0.588   -0.125   -0.045   -0.298    STW_ttl (.11.)    0.216    0.041    5.330    0.000    0.136    0.295    0.027    0.496    Cttll.t (.12.)    0.255    0.048    5.292    0.000    0.160    0.349    0.032    0.547    smmbV_N (.13.)   -0.055    0.063   -0.871    0.384   -0.179    0.069   -0.007   -0.062    Fcs_FAM (.14.)    0.083    0.029    2.851    0.004    0.026    0.140    0.011    0.179Regressions:                   Estimate  Std.Err  z-value  P(>|z|) ci.lower ci.upper   Std.lv  Std.all  factor ~                                                                                    meRLC   (LC__)    0.921    0.331    2.784    0.005    0.273    1.569    7.298    0.216Intercepts:                   Estimate  Std.Err  z-value  P(>|z|) ci.lower ci.upper   Std.lv  Std.all   .EMoneg            0.566    0.037   15.106    0.000    0.493    0.639    0.566    3.321   .EMvneg            0.269    0.041    6.612    0.000    0.189    0.349    0.269    1.336   .EMpneg            0.128    0.012   10.491    0.000    0.104    0.151    0.128    1.068   .ERneg             0.428    0.029   14.498    0.000    0.370    0.485    0.428    1.909   .ERnegreac         0.020    0.002    8.326    0.000    0.015    0.024    0.020    0.957   .SSRTb             0.172    0.011   15.672    0.000    0.151    0.194    0.172    3.496   .Hoteltask_Time    0.389    0.018   21.147    0.000    0.353    0.425    0.389    2.090   .PSQI_score        0.063    0.004   14.146    0.000    0.055    0.072    0.063    1.623   .education_age     0.180    0.007   27.301    0.000    0.167    0.193    0.180    3.954   .Occ_score         0.253    0.020   12.873    0.000    0.215    0.292    0.253    1.676   .STW_total         0.525    0.009   61.243    0.000    0.509    0.542    0.525    9.563   .Cattell.ttlscr    0.252    0.009   26.623    0.000    0.233    0.271    0.252    4.293   .semmbVsmnmb_pN    0.199    0.009   21.323    0.000    0.181    0.218    0.199    1.768   .Faces_FAMnam      0.201    0.005   40.151    0.000    0.191    0.211    0.201    3.420   .factor            0.000                               0.000    0.000    0.000    0.000Variances:                   Estimate  Std.Err  z-value  P(>|z|) ci.lower ci.upper   Std.lv  Std.all   .EMoneg            0.013    0.003    4.879    0.000    0.008    0.018    0.013    0.452   .EMvneg            0.017    0.003    5.366    0.000    0.011    0.023    0.017    0.411   .EMpneg            0.014    0.002    9.160    0.000    0.011    0.017    0.014    0.995   .ERneg             0.049    0.010    4.841    0.000    0.029    0.069    0.049    0.974   .ERnegreac         0.000    0.000    6.347    0.000    0.000    0.001    0.000    0.997   .SSRTb             0.002    0.000    5.551    0.000    0.002    0.003    0.002    0.958   .Hoteltask_Time    0.031    0.003    9.557    0.000    0.025    0.038    0.031    0.908   .PSQI_score        0.001    0.000    8.859    0.000    0.001    0.002    0.001    0.955   .education_age     0.002    0.000    4.254    0.000    0.001    0.002    0.002    0.737   .Occ_score         0.021    0.003    6.594    0.000    0.015    0.027    0.021    0.911   .STW_total         0.002    0.000    5.265    0.000    0.001    0.003    0.002    0.754   .Cattell.ttlscr    0.002    0.000    8.085    0.000    0.002    0.003    0.002    0.701   .semmbVsmnmb_pN    0.013    0.002    8.003    0.000    0.010    0.016    0.013    0.996   .Faces_FAMnam      0.003    0.000   13.120    0.000    0.003    0.004    0.003    0.968   .factor            0.015    0.004    4.024    0.000    0.008    0.023    0.953    0.953R-Square:                   Estimate    EMoneg            0.548    EMvneg            0.589    EMpneg            0.005    ERneg             0.026    ERnegreac         0.003    SSRTb             0.042    Hoteltask_Time    0.092    PSQI_score        0.045    education_age     0.263    Occ_score         0.089    STW_total         0.246    Cattell.ttlscr    0.299    semmbVsmnmb_pN    0.004    Faces_FAMnam      0.032    factor            0.047Group 2 [younger]:Latent Variables:                   Estimate  Std.Err  z-value  P(>|z|) ci.lower ci.upper   Std.lv  Std.all  factor =~                                                                                   EMoneg            1.000                               1.000    1.000    0.084    0.690    EMvneg  (.p2.)    1.224    0.128    9.603    0.000    0.975    1.474    0.103    0.644    EMpneg  (.p3.)    0.066    0.075    0.878    0.380   -0.081    0.212    0.006    0.054    ERneg   (.p4.)    0.284    0.212    1.340    0.180   -0.131    0.699    0.024    0.139    ERnegrc (.p5.)   -0.009    0.019   -0.476    0.634   -0.046    0.028   -0.001   -0.044    SSRTb   (.p6.)    0.080    0.073    1.087    0.277   -0.064    0.223    0.007    0.164    Htlts_T (.p7.)   -0.449    0.102   -4.385    0.000   -0.649   -0.248   -0.038   -0.251    PSQI_sc (.p8.)   -0.066    0.026   -2.488    0.013   -0.117   -0.014   -0.006   -0.153    edctn_g (.p9.)    0.185    0.034    5.428    0.000    0.118    0.252    0.016    0.479    Occ_scr (.10.)   -0.356    0.118   -3.017    0.003   -0.588   -0.125   -0.030   -0.185    STW_ttl (.11.)    0.216    0.041    5.330    0.000    0.136    0.295    0.018    0.351    Cttll.t (.12.)    0.255    0.048    5.292    0.000    0.160    0.349    0.021    0.473    smmbV_N (.13.)   -0.055    0.063   -0.871    0.384   -0.179    0.069   -0.005   -0.046    Fcs_FAM (.14.)    0.083    0.029    2.851    0.004    0.026    0.140    0.007    0.155Regressions:                   Estimate  Std.Err  z-value  P(>|z|) ci.lower ci.upper   Std.lv  Std.all  factor ~                                                                                    meRLC   (LC__)   -0.562    0.247   -2.277    0.023   -1.046   -0.078   -6.670   -0.164Intercepts:                   Estimate  Std.Err  z-value  P(>|z|) ci.lower ci.upper   Std.lv  Std.all   .EMoneg            0.888    0.023   38.593    0.000    0.843    0.933    0.888    7.265   .EMvneg            0.710    0.030   23.572    0.000    0.651    0.769    0.710    4.434   .EMpneg            0.176    0.009   18.934    0.000    0.157    0.194    0.176    1.728   .ERneg             0.556    0.016   33.948    0.000    0.524    0.588    0.556    3.234   .ERnegreac         0.030    0.002   19.278    0.000    0.027    0.033    0.030    1.749   .SSRTb             0.171    0.006   26.373    0.000    0.158    0.184    0.171    4.173   .Hoteltask_Time    0.241    0.013   18.524    0.000    0.216    0.267    0.241    1.601   .PSQI_score        0.048    0.002   19.714    0.000    0.043    0.053    0.048    1.335   .education_age     0.223    0.004   54.321    0.000    0.215    0.231    0.223    6.842   .Occ_score         0.192    0.011   17.118    0.000    0.170    0.213    0.192    1.178   .STW_total         0.541    0.005  109.449    0.000    0.531    0.551    0.541   10.451   .Cattell.ttlscr    0.370    0.006   60.912    0.000    0.358    0.382    0.370    8.153   .semmbVsmnmb_pN    0.177    0.006   28.091    0.000    0.165    0.190    0.177    1.743   .Faces_FAMnam      0.262    0.003   81.450    0.000    0.256    0.269    0.262    5.801   .factor            0.000                               0.000    0.000    0.000    0.000Variances:                   Estimate  Std.Err  z-value  P(>|z|) ci.lower ci.upper   Std.lv  Std.all   .EMoneg            0.008    0.002    4.522    0.000    0.004    0.011    0.008    0.524   .EMvneg            0.015    0.003    4.978    0.000    0.009    0.021    0.015    0.585   .EMpneg            0.010    0.001   10.418    0.000    0.008    0.012    0.010    0.997   .ERneg             0.029    0.003    9.218    0.000    0.023    0.035    0.029    0.981   .ERnegreac         0.000    0.000    8.697    0.000    0.000    0.000    0.000    0.998   .SSRTb             0.002    0.000    7.004    0.000    0.001    0.002    0.002    0.973   .Hoteltask_Time    0.021    0.002   11.570    0.000    0.018    0.025    0.021    0.937   .PSQI_score        0.001    0.000    8.415    0.000    0.001    0.002    0.001    0.977   .education_age     0.001    0.000    8.129    0.000    0.001    0.001    0.001    0.771   .Occ_score         0.026    0.003    7.979    0.000    0.019    0.032    0.026    0.966   .STW_total         0.002    0.000    6.038    0.000    0.002    0.003    0.002    0.876   .Cattell.ttlscr    0.002    0.000   11.193    0.000    0.001    0.002    0.002    0.776   .semmbVsmnmb_pN    0.010    0.001   12.822    0.000    0.009    0.012    0.010    0.998   .Faces_FAMnam      0.002    0.000    8.398    0.000    0.002    0.002    0.002    0.976   .factor            0.007    0.002    3.201    0.001    0.003    0.011    0.973    0.973R-Square:                   Estimate    EMoneg            0.476    EMvneg            0.415    EMpneg            0.003    ERneg             0.019    ERnegreac         0.002    SSRTb             0.027    Hoteltask_Time    0.063    PSQI_score        0.023    education_age     0.229    Occ_score         0.034    STW_total         0.124    Cattell.ttlscr    0.224    semmbVsmnmb_pN    0.002    Faces_FAMnam      0.024    factor            0.027*AGE-ADJUSTED MULTI-GROUP UNIDIMENSIONAL MODEL - ROSTRAL LC> unconstrained.model.rostral<-'+ factor=~ EMoneg+EMvneg +EMpneg+ERneg + ERnegreac +SSRTb+ Hoteltask_Time+ PSQI_score+education_age+Occ_score+STW_total+Cattell.totalscore+semambVsemunamb_pNo + Faces_FAMnam + factor ~ c(LC_effect_young,LC_effect_old)*Rmeanhigh+ factor~age_years+ Rmeanhigh~age_years'> unconstrained.model.rostral.fit<- cfa(unconstrained.model.rostral, data=ourdata1,  group="agecat",group.equal = c("loadings"),missing = "ML", estimator='mlr')Warning messages:1: In lav_data_full(data = data, group = group, cluster = cluster,  :  lavaan WARNING: due to missing values, some pairwise combinations have less than 10% coverage2: In lav_data_full(data = data, group = group, cluster = cluster,  :  lavaan WARNING: due to missing values, some pairwise combinations have less than 10% coverage> summary(unconstrained.model.rostral.fit,fit.measures=TRUE, standardized=TRUE, rsquare=TRUE, ci=TRUE)lavaan 0.6-3 ended normally after 348 iterations  Optimization method                           NLMINB  Number of free parameters                         94  Number of equality constraints                    13  Number of observations per group           older                                            269  younger                                          336  Number of missing patterns per group       older                                             25  younger                                           24  Estimator                                         ML      Robust  Model Fit Test Statistic                     622.564     631.866  Degrees of freedom                               219         219  P-value (Chi-square)                           0.000       0.000  Scaling correction factor                                  0.985    for the Yuan-Bentler correction (Mplus variant)Chi-square for each group:  older                                        264.663     268.617  younger                                      357.902     363.249User model versus baseline model:  Comparative Fit Index (CFI)                       NA          NA  Tucker-Lewis Index (TLI)                          NA          NA  Robust Comparative Fit Index (CFI)                            NA  Robust Tucker-Lewis Index (TLI)                               NALoglikelihood and Information Criteria:  Loglikelihood user model (H0)               8842.737    8842.737  Scaling correction factor                                  1.119    for the MLR correction  Loglikelihood unrestricted model (H1)       9154.019    9154.019  Scaling correction factor                                  1.092    for the MLR correction  Number of free parameters                         81          81  Akaike (AIC)                              -17523.474  -17523.474  Bayesian (BIC)                            -17166.651  -17166.651  Sample-size adjusted Bayesian (BIC)       -17423.806  -17423.806Root Mean Square Error of Approximation:  RMSEA                                          0.078       0.079  90 Percent Confidence Interval          0.071  0.085       0.072  0.086  P-value RMSEA <= 0.05                          0.000       0.000  Robust RMSEA                                               0.078  90 Percent Confidence Interval                             0.071  0.086Standardized Root Mean Square Residual:  SRMR                                           0.118       0.118Parameter Estimates:  Information                                 Observed  Observed information based on                Hessian  Standard Errors                   Robust.huber.whiteGroup 1 [older]:Latent Variables:                   Estimate  Std.Err  z-value  P(>|z|) ci.lower ci.upper   Std.lv  Std.all  factor =~                                                                                   EMoneg            1.000                               1.000    1.000    0.128    0.742    EMvneg  (.p2.)    1.259    0.133    9.436    0.000    0.997    1.520    0.161    0.790    EMpneg  (.p3.)    0.082    0.072    1.145    0.252   -0.059    0.224    0.011    0.088    ERneg   (.p4.)    0.465    0.191    2.438    0.015    0.091    0.840    0.060    0.269    ERnegrc (.p5.)    0.020    0.020    0.978    0.328   -0.020    0.059    0.003    0.125    SSRTb   (.p6.)    0.034    0.071    0.484    0.628   -0.104    0.173    0.004    0.091    Htlts_T (.p7.)   -0.465    0.098   -4.720    0.000   -0.658   -0.272   -0.060   -0.320    PSQI_sc (.p8.)   -0.057    0.025   -2.236    0.025   -0.106   -0.007   -0.007   -0.186    edctn_g (.p9.)    0.160    0.031    5.101    0.000    0.099    0.222    0.021    0.446    Occ_scr (.10.)   -0.232    0.118   -1.973    0.048   -0.463   -0.002   -0.030   -0.197    STW_ttl (.11.)    0.151    0.043    3.531    0.000    0.067    0.234    0.019    0.350    Cttll.t (.12.)    0.308    0.054    5.749    0.000    0.203    0.413    0.039    0.668    smmbV_N (.13.)   -0.035    0.060   -0.581    0.561   -0.152    0.083   -0.004   -0.040    Fcs_FAM (.14.)    0.110    0.034    3.264    0.001    0.044    0.177    0.014    0.245Regressions:                   Estimate  Std.Err  z-value  P(>|z|) ci.lower ci.upper   Std.lv  Std.all  factor ~                                                                                    Rmenhgh (LC__)    0.733    0.297    2.468    0.014    0.151    1.315    5.727    0.194    age_yrs          -0.790    0.147   -5.361    0.000   -1.079   -0.501   -6.170   -0.501  Rmeanhigh ~                                                                                 age_yrs          -0.042    0.024   -1.771    0.077   -0.089    0.005   -0.042   -0.102Intercepts:                   Estimate  Std.Err  z-value  P(>|z|) ci.lower ci.upper   Std.lv  Std.all   .EMoneg            1.141    0.116    9.800    0.000    0.913    1.369    1.141    6.610   .EMvneg            0.990    0.152    6.519    0.000    0.692    1.287    0.990    4.849   .EMpneg            0.173    0.039    4.425    0.000    0.097    0.250    0.173    1.452   .ERneg             0.679    0.122    5.577    0.000    0.440    0.918    0.679    3.062   .ERnegreac         0.028    0.012    2.416    0.016    0.005    0.051    0.028    1.405   .SSRTb             0.197    0.033    6.042    0.000    0.133    0.261    0.197    4.078   .Hoteltask_Time    0.124    0.067    1.842    0.066   -0.008    0.255    0.124    0.666   .PSQI_score        0.030    0.011    2.828    0.005    0.009    0.051    0.030    0.772   .education_age     0.274    0.017   16.311    0.000    0.241    0.307    0.274    5.961   .Occ_score         0.108    0.048    2.259    0.024    0.014    0.202    0.108    0.717   .STW_total         0.618    0.016   38.335    0.000    0.586    0.649    0.618   11.201   .Cattell.ttlscr    0.424    0.044    9.720    0.000    0.339    0.510    0.424    7.185   .semmbVsmnmb_pN    0.177    0.029    6.169    0.000    0.121    0.234    0.177    1.575   .Faces_FAMnam      0.262    0.023   11.289    0.000    0.216    0.307    0.262    4.533   .Rmeanhigh         0.143    0.017    8.310    0.000    0.109    0.177    0.143    4.232   .factor            0.000                               0.000    0.000    0.000    0.000Variances:                   Estimate  Std.Err  z-value  P(>|z|) ci.lower ci.upper   Std.lv  Std.all   .EMoneg            0.013    0.002    5.742    0.000    0.009    0.018    0.013    0.450   .EMvneg            0.016    0.003    5.637    0.000    0.010    0.021    0.016    0.376   .EMpneg            0.014    0.002    9.149    0.000    0.011    0.017    0.014    0.992   .ERneg             0.046    0.010    4.561    0.000    0.026    0.065    0.046    0.928   .ERnegreac         0.000    0.000    6.780    0.000    0.000    0.001    0.000    0.984   .SSRTb             0.002    0.000    5.946    0.000    0.002    0.003    0.002    0.992   .Hoteltask_Time    0.031    0.003    9.513    0.000    0.025    0.037    0.031    0.897   .PSQI_score        0.001    0.000    8.811    0.000    0.001    0.002    0.001    0.965   .education_age     0.002    0.000    4.583    0.000    0.001    0.002    0.002    0.801   .Occ_score         0.022    0.003    7.066    0.000    0.016    0.028    0.022    0.961   .STW_total         0.003    0.000    5.923    0.000    0.002    0.004    0.003    0.878   .Cattell.ttlscr    0.002    0.000    7.088    0.000    0.001    0.002    0.002    0.554   .semmbVsmnmb_pN    0.013    0.002    8.025    0.000    0.010    0.016    0.013    0.998   .Faces_FAMnam      0.003    0.000   11.711    0.000    0.003    0.004    0.003    0.940   .Rmeanhigh         0.001    0.000    9.741    0.000    0.001    0.001    0.001    0.990   .factor            0.011    0.003    3.861    0.000    0.006    0.017    0.692    0.692R-Square:                   Estimate    EMoneg            0.550    EMvneg            0.624    EMpneg            0.008    ERneg             0.072    ERnegreac         0.016    SSRTb             0.008    Hoteltask_Time    0.103    PSQI_score        0.035    education_age     0.199    Occ_score         0.039    STW_total         0.122    Cattell.ttlscr    0.446    semmbVsmnmb_pN    0.002    Faces_FAMnam      0.060    Rmeanhigh         0.010    factor            0.308Group 2 [younger]:Latent Variables:                   Estimate  Std.Err  z-value  P(>|z|) ci.lower ci.upper   Std.lv  Std.all  factor =~                                                                                   EMoneg            1.000                               1.000    1.000    0.078    0.661    EMvneg  (.p2.)    1.259    0.133    9.436    0.000    0.997    1.520    0.099    0.634    EMpneg  (.p3.)    0.082    0.072    1.145    0.252   -0.059    0.224    0.006    0.063    ERneg   (.p4.)    0.465    0.191    2.438    0.015    0.091    0.840    0.036    0.210    ERnegrc (.p5.)    0.020    0.020    0.978    0.328   -0.020    0.059    0.002    0.088    SSRTb   (.p6.)    0.034    0.071    0.484    0.628   -0.104    0.173    0.003    0.065    Htlts_T (.p7.)   -0.465    0.098   -4.720    0.000   -0.658   -0.272   -0.036   -0.241    PSQI_sc (.p8.)   -0.057    0.025   -2.236    0.025   -0.106   -0.007   -0.004   -0.122    edctn_g (.p9.)    0.160    0.031    5.101    0.000    0.099    0.222    0.013    0.389    Occ_scr (.10.)   -0.232    0.118   -1.973    0.048   -0.463   -0.002   -0.018   -0.112    STW_ttl (.11.)    0.151    0.043    3.531    0.000    0.067    0.234    0.012    0.229    Cttll.t (.12.)    0.308    0.054    5.749    0.000    0.203    0.413    0.024    0.533    smmbV_N (.13.)   -0.035    0.060   -0.581    0.561   -0.152    0.083   -0.003   -0.027    Fcs_FAM (.14.)    0.110    0.034    3.264    0.001    0.044    0.177    0.009    0.188Regressions:                   Estimate  Std.Err  z-value  P(>|z|) ci.lower ci.upper   Std.lv  Std.all  factor ~                                                                                    Rmenhgh (LC__)   -0.073    0.228   -0.322    0.748   -0.521    0.374   -0.939   -0.025    age_yrs          -0.218    0.080   -2.718    0.007   -0.375   -0.061   -2.785   -0.288  Rmeanhigh ~                                                                                 age_yrs           0.101    0.014    7.198    0.000    0.073    0.128    0.101    0.389Intercepts:                   Estimate  Std.Err  z-value  P(>|z|) ci.lower ci.upper   Std.lv  Std.all   .EMoneg            0.935    0.031   30.610    0.000    0.875    0.995    0.935    7.897   .EMvneg            0.771    0.042   18.537    0.000    0.689    0.852    0.771    4.962   .EMpneg            0.180    0.011   15.968    0.000    0.158    0.202    0.180    1.773   .ERneg             0.585    0.025   23.316    0.000    0.536    0.634    0.585    3.370   .ERnegreac         0.033    0.002   13.595    0.000    0.028    0.037    0.033    1.856   .SSRTb             0.171    0.008   20.601    0.000    0.154    0.187    0.171    4.127   .Hoteltask_Time    0.219    0.017   13.221    0.000    0.187    0.251    0.219    1.450   .PSQI_score        0.046    0.003   15.839    0.000    0.040    0.052    0.046    1.272   .education_age     0.229    0.005   48.277    0.000    0.220    0.238    0.229    7.098   .Occ_score         0.187    0.012   15.084    0.000    0.163    0.211    0.187    1.151   .STW_total         0.545    0.005  116.132    0.000    0.536    0.554    0.545   10.564   .Cattell.ttlscr    0.387    0.010   37.836    0.000    0.367    0.407    0.387    8.538   .semmbVsmnmb_pN    0.177    0.008   23.065    0.000    0.162    0.192    0.177    1.736   .Faces_FAMnam      0.269    0.005   58.531    0.000    0.260    0.278    0.269    5.855   .Rmeanhigh         0.064    0.005   11.717    0.000    0.053    0.074    0.064    2.367   .factor            0.000                               0.000    0.000    0.000    0.000Variances:                   Estimate  Std.Err  z-value  P(>|z|) ci.lower ci.upper   Std.lv  Std.all   .EMoneg            0.008    0.002    4.807    0.000    0.005    0.011    0.008    0.562   .EMvneg            0.014    0.003    4.938    0.000    0.009    0.020    0.014    0.598   .EMpneg            0.010    0.001   10.401    0.000    0.008    0.012    0.010    0.996   .ERneg             0.029    0.003    9.094    0.000    0.023    0.035    0.029    0.956   .ERnegreac         0.000    0.000    8.896    0.000    0.000    0.000    0.000    0.992   .SSRTb             0.002    0.000    7.170    0.000    0.001    0.002    0.002    0.996   .Hoteltask_Time    0.021    0.002   11.508    0.000    0.018    0.025    0.021    0.942   .PSQI_score        0.001    0.000    8.313    0.000    0.001    0.002    0.001    0.985   .education_age     0.001    0.000    8.686    0.000    0.001    0.001    0.001    0.849   .Occ_score         0.026    0.003    7.845    0.000    0.020    0.033    0.026    0.987   .STW_total         0.003    0.000    6.225    0.000    0.002    0.003    0.003    0.948   .Cattell.ttlscr    0.001    0.000    9.246    0.000    0.001    0.002    0.001    0.716   .semmbVsmnmb_pN    0.010    0.001   12.897    0.000    0.009    0.012    0.010    0.999   .Faces_FAMnam      0.002    0.000    8.296    0.000    0.002    0.003    0.002    0.965   .Rmeanhigh         0.001    0.000   11.074    0.000    0.001    0.001    0.001    0.849   .factor            0.006    0.002    2.937    0.003    0.002    0.009    0.911    0.911R-Square:                   Estimate    EMoneg            0.438    EMvneg            0.402    EMpneg            0.004    ERneg             0.044    ERnegreac         0.008    SSRTb             0.004    Hoteltask_Time    0.058    PSQI_score        0.015    education_age     0.151    Occ_score         0.013    STW_total         0.052    Cattell.ttlscr    0.284    semmbVsmnmb_pN    0.001    Faces_FAMnam      0.035    Rmeanhigh         0.151    factor            0.089*NON AGE-ADJUSTED MULTIGROUP UNIDIMENSIONAL MODEL - ROSTRAL LC> unconstrained.rostral.noage<-'+ factor=~ EMoneg+EMvneg +EMpneg+ERneg + ERnegreac +SSRTb+ Hoteltask_Time+ PSQI_score+education_age+Occ_score+STW_total+Cattell.totalscore+semambVsemunamb_pNo + Faces_FAMnam + factor ~ c(LC_effect_young,LC_effect_old)*Rmeanhigh'> unconstrained.rostral.noage.fit<- cfa(unconstrained.rostral.noage, data=ourdata1,  group="agecat",group.equal = c("loadings"),missing = "ML", estimator='mlr')Warning messages:1: In lav_data_full(data = data, group = group, cluster = cluster,  :  lavaan WARNING: due to missing values, some pairwise combinations have less than 10% coverage2: In lav_data_full(data = data, group = group, cluster = cluster,  :  lavaan WARNING: due to missing values, some pairwise combinations have less than 10% coverage> summary(unconstrained.rostral.noage.fit,fit.measures=TRUE, standardized=TRUE, rsquare=TRUE, ci=TRUE)lavaan 0.6-3 ended normally after 342 iterations  Optimization method                           NLMINB  Number of free parameters                         86  Number of equality constraints                    13  Number of observations per group           older                                            269  younger                                          336  Number of missing patterns per group       older                                             25  younger                                           24  Estimator                                         ML      Robust  Model Fit Test Statistic                     378.848     392.586  Degrees of freedom                               193         193  P-value (Chi-square)                           0.000       0.000  Scaling correction factor                                  0.965    for the Yuan-Bentler correction (Mplus variant)Chi-square for each group:  older                                        155.294     160.925  younger                                      223.554     231.661User model versus baseline model:  Comparative Fit Index (CFI)                       NA          NA  Tucker-Lewis Index (TLI)                          NA          NA  Robust Comparative Fit Index (CFI)                            NA  Robust Tucker-Lewis Index (TLI)                               NALoglikelihood and Information Criteria:  Loglikelihood user model (H0)               7520.313    7520.313  Scaling correction factor                                  1.127    for the MLR correction  Loglikelihood unrestricted model (H1)       7709.737    7709.737  Scaling correction factor                                  1.089    for the MLR correction  Number of free parameters                         73          73  Akaike (AIC)                              -14894.626  -14894.626  Bayesian (BIC)                            -14573.045  -14573.045  Sample-size adjusted Bayesian (BIC)       -14804.802  -14804.802Root Mean Square Error of Approximation:  RMSEA                                          0.056       0.058  90 Percent Confidence Interval          0.048  0.065       0.050  0.067  P-value RMSEA <= 0.05                          0.103       0.050  Robust RMSEA                                               0.057  90 Percent Confidence Interval                             0.049  0.066Standardized Root Mean Square Residual:  SRMR                                           0.117       0.117Parameter Estimates:  Information                                 Observed  Observed information based on                Hessian  Standard Errors                   Robust.huber.whiteGroup 1 [older]:Latent Variables:                   Estimate  Std.Err  z-value  P(>|z|) ci.lower ci.upper   Std.lv  Std.all  factor =~                                                                                   EMoneg            1.000                               1.000    1.000    0.126    0.738    EMvneg  (.p2.)    1.227    0.127    9.650    0.000    0.977    1.476    0.154    0.767    EMpneg  (.p3.)    0.067    0.075    0.888    0.375   -0.080    0.214    0.008    0.070    ERneg   (.p4.)    0.287    0.212    1.352    0.176   -0.129    0.703    0.036    0.161    ERnegrc (.p5.)   -0.010    0.019   -0.543    0.587   -0.047    0.027   -0.001   -0.063    SSRTb   (.p6.)    0.080    0.074    1.077    0.282   -0.066    0.226    0.010    0.204    Htlts_T (.p7.)   -0.447    0.102   -4.359    0.000   -0.648   -0.246   -0.056   -0.301    PSQI_sc (.p8.)   -0.067    0.027   -2.488    0.013   -0.119   -0.014   -0.008   -0.214    edctn_g (.p9.)    0.186    0.034    5.397    0.000    0.118    0.253    0.023    0.513    Occ_scr (.10.)   -0.363    0.119   -3.053    0.002   -0.596   -0.130   -0.046   -0.302    STW_ttl (.11.)    0.218    0.041    5.319    0.000    0.138    0.299    0.027    0.498    Cttll.t (.12.)    0.254    0.048    5.285    0.000    0.160    0.348    0.032    0.543    smmbV_N (.13.)   -0.053    0.063   -0.835    0.403   -0.177    0.071   -0.007   -0.059    Fcs_FAM (.14.)    0.083    0.029    2.831    0.005    0.026    0.141    0.010    0.178Regressions:                   Estimate  Std.Err  z-value  P(>|z|) ci.lower ci.upper   Std.lv  Std.all  factor ~                                                                                    Rmenhgh (LC__)    0.969    0.315    3.078    0.002    0.352    1.587    7.721    0.261Intercepts:                   Estimate  Std.Err  z-value  P(>|z|) ci.lower ci.upper   Std.lv  Std.all   .EMoneg            0.548    0.040   13.709    0.000    0.470    0.626    0.548    3.219   .EMvneg            0.247    0.045    5.541    0.000    0.159    0.334    0.247    1.229   .EMpneg            0.126    0.013    9.739    0.000    0.101    0.152    0.126    1.057   .ERneg             0.422    0.032   13.042    0.000    0.359    0.486    0.422    1.885   .ERnegreac         0.020    0.003    7.652    0.000    0.015    0.025    0.020    0.971   .SSRTb             0.171    0.012   14.288    0.000    0.147    0.194    0.171    3.465   .Hoteltask_Time    0.397    0.020   20.015    0.000    0.358    0.436    0.397    2.133   .PSQI_score        0.065    0.005   13.291    0.000    0.055    0.074    0.065    1.654   .education_age     0.177    0.007   25.113    0.000    0.163    0.190    0.177    3.880   .Occ_score         0.260    0.021   12.295    0.000    0.219    0.302    0.260    1.722   .STW_total         0.521    0.009   58.059    0.000    0.504    0.539    0.521    9.477   .Cattell.ttlscr    0.248    0.010   24.634    0.000    0.228    0.267    0.248    4.222   .semmbVsmnmb_pN    0.200    0.010   19.864    0.000    0.180    0.220    0.200    1.775   .Faces_FAMnam      0.199    0.005   37.500    0.000    0.189    0.210    0.199    3.394   .factor            0.000                               0.000    0.000    0.000    0.000Variances:                   Estimate  Std.Err  z-value  P(>|z|) ci.lower ci.upper   Std.lv  Std.all   .EMoneg            0.013    0.003    4.931    0.000    0.008    0.018    0.013    0.456   .EMvneg            0.017    0.003    5.390    0.000    0.011    0.023    0.017    0.411   .EMpneg            0.014    0.002    9.163    0.000    0.011    0.017    0.014    0.995   .ERneg             0.049    0.010    4.860    0.000    0.029    0.069    0.049    0.974   .ERnegreac         0.000    0.000    6.325    0.000    0.000    0.001    0.000    0.996   .SSRTb             0.002    0.000    5.563    0.000    0.002    0.003    0.002    0.958   .Hoteltask_Time    0.032    0.003    9.574    0.000    0.025    0.038    0.032    0.909   .PSQI_score        0.001    0.000    8.853    0.000    0.001    0.002    0.001    0.954   .education_age     0.002    0.000    4.269    0.000    0.001    0.002    0.002    0.737   .Occ_score         0.021    0.003    6.602    0.000    0.015    0.027    0.021    0.909   .STW_total         0.002    0.000    5.270    0.000    0.001    0.003    0.002    0.752   .Cattell.ttlscr    0.002    0.000    8.102    0.000    0.002    0.003    0.002    0.705   .semmbVsmnmb_pN    0.013    0.002    8.005    0.000    0.010    0.016    0.013    0.997   .Faces_FAMnam      0.003    0.000   13.135    0.000    0.003    0.004    0.003    0.968   .factor            0.015    0.004    3.966    0.000    0.007    0.022    0.932    0.932R-Square:                   Estimate    EMoneg            0.544    EMvneg            0.589    EMpneg            0.005    ERneg             0.026    ERnegreac         0.004    SSRTb             0.042    Hoteltask_Time    0.091    PSQI_score        0.046    education_age     0.263    Occ_score         0.091    STW_total         0.248    Cattell.ttlscr    0.295    semmbVsmnmb_pN    0.003    Faces_FAMnam      0.032    factor            0.068Group 2 [younger]:Latent Variables:                   Estimate  Std.Err  z-value  P(>|z|) ci.lower ci.upper   Std.lv  Std.all  factor =~                                                                                   EMoneg            1.000                               1.000    1.000    0.084    0.688    EMvneg  (.p2.)    1.227    0.127    9.650    0.000    0.977    1.476    0.103    0.643    EMpneg  (.p3.)    0.067    0.075    0.888    0.375   -0.080    0.214    0.006    0.055    ERneg   (.p4.)    0.287    0.212    1.352    0.176   -0.129    0.703    0.024    0.140    ERnegrc (.p5.)   -0.010    0.019   -0.543    0.587   -0.047    0.027   -0.001   -0.049    SSRTb   (.p6.)    0.080    0.074    1.077    0.282   -0.066    0.226    0.007    0.164    Htlts_T (.p7.)   -0.447    0.102   -4.359    0.000   -0.648   -0.246   -0.038   -0.249    PSQI_sc (.p8.)   -0.067    0.027   -2.488    0.013   -0.119   -0.014   -0.006   -0.155    edctn_g (.p9.)    0.186    0.034    5.397    0.000    0.118    0.253    0.016    0.480    Occ_scr (.10.)   -0.363    0.119   -3.053    0.002   -0.596   -0.130   -0.030   -0.187    STW_ttl (.11.)    0.218    0.041    5.319    0.000    0.138    0.299    0.018    0.355    Cttll.t (.12.)    0.254    0.048    5.285    0.000    0.160    0.348    0.021    0.470    smmbV_N (.13.)   -0.053    0.063   -0.835    0.403   -0.177    0.071   -0.004   -0.044    Fcs_FAM (.14.)    0.083    0.029    2.831    0.005    0.026    0.141    0.007    0.154Regressions:                   Estimate  Std.Err  z-value  P(>|z|) ci.lower ci.upper   Std.lv  Std.all  factor ~                                                                                    Rmenhgh (LC__)   -0.341    0.229   -1.490    0.136   -0.789    0.107   -4.053   -0.109Intercepts:                   Estimate  Std.Err  z-value  P(>|z|) ci.lower ci.upper   Std.lv  Std.all   .EMoneg            0.875    0.025   35.022    0.000    0.826    0.923    0.875    7.160   .EMvneg            0.694    0.032   21.685    0.000    0.631    0.757    0.694    4.329   .EMpneg            0.175    0.009   19.821    0.000    0.157    0.192    0.175    1.719   .ERneg             0.552    0.016   34.435    0.000    0.521    0.584    0.552    3.212   .ERnegreac         0.030    0.001   20.651    0.000    0.028    0.033    0.030    1.754   .SSRTb             0.170    0.006   27.719    0.000    0.158    0.182    0.170    4.148   .Hoteltask_Time    0.247    0.013   18.641    0.000    0.221    0.273    0.247    1.640   .PSQI_score        0.049    0.002   19.790    0.000    0.044    0.054    0.049    1.359   .education_age     0.220    0.004   49.657    0.000    0.212    0.229    0.220    6.764   .Occ_score         0.196    0.012   16.882    0.000    0.173    0.219    0.196    1.206   .STW_total         0.538    0.005   98.190    0.000    0.528    0.549    0.538   10.409   .Cattell.ttlscr    0.366    0.006   57.244    0.000    0.354    0.379    0.366    8.068   .semmbVsmnmb_pN    0.178    0.006   29.579    0.000    0.166    0.190    0.178    1.751   .Faces_FAMnam      0.261    0.003   80.976    0.000    0.255    0.268    0.261    5.778   .factor            0.000                               0.000    0.000    0.000    0.000Variances:                   Estimate  Std.Err  z-value  P(>|z|) ci.lower ci.upper   Std.lv  Std.all   .EMoneg            0.008    0.002    4.477    0.000    0.004    0.011    0.008    0.527   .EMvneg            0.015    0.003    4.963    0.000    0.009    0.021    0.015    0.586   .EMpneg            0.010    0.001   10.419    0.000    0.008    0.012    0.010    0.997   .ERneg             0.029    0.003    9.215    0.000    0.023    0.035    0.029    0.980   .ERnegreac         0.000    0.000    8.685    0.000    0.000    0.000    0.000    0.998   .SSRTb             0.002    0.000    6.991    0.000    0.001    0.002    0.002    0.973   .Hoteltask_Time    0.021    0.002   11.575    0.000    0.018    0.025    0.021    0.938   .PSQI_score        0.001    0.000    8.426    0.000    0.001    0.002    0.001    0.976   .education_age     0.001    0.000    8.139    0.000    0.001    0.001    0.001    0.770   .Occ_score         0.026    0.003    7.984    0.000    0.019    0.032    0.026    0.965   .STW_total         0.002    0.000    6.002    0.000    0.002    0.003    0.002    0.874   .Cattell.ttlscr    0.002    0.000   11.283    0.000    0.001    0.002    0.002    0.779   .semmbVsmnmb_pN    0.010    0.001   12.826    0.000    0.009    0.012    0.010    0.998   .Faces_FAMnam      0.002    0.000    8.400    0.000    0.002    0.002    0.002    0.976   .factor            0.007    0.002    3.219    0.001    0.003    0.011    0.988    0.988R-Square:                   Estimate    EMoneg            0.473    EMvneg            0.414    EMpneg            0.003    ERneg             0.020    ERnegreac         0.002    SSRTb             0.027    Hoteltask_Time    0.062    PSQI_score        0.024    education_age     0.230    Occ_score         0.035    STW_total         0.126    Cattell.ttlscr    0.221    semmbVsmnmb_pN    0.002    Faces_FAMnam      0.024    factor            0.012*AGE-ADJUSTED MULTIGROUP UNIDIMENSIONAL MODEL - CAUDAL LC> unconstrained.model.caudal<-'+ factor=~ EMoneg+EMvneg +EMpneg+ERneg + ERnegreac +SSRTb+ Hoteltask_Time+ PSQI_score+education_age+Occ_score+STW_total+Cattell.totalscore+semambVsemunamb_pNo + Faces_FAMnam + factor ~ c(LC_effect_young,LC_effect_old)*Rmeanlow+ factor~age_years+ Rmeanlow~age_years'> unconstrained.model.caudal.fit<- cfa(unconstrained.model.caudal, data=ourdata1,  group="agecat",group.equal = c("loadings"),missing = "ML", estimator='mlr')Warning messages:1: In lav_data_full(data = data, group = group, cluster = cluster,  :  lavaan WARNING: due to missing values, some pairwise combinations have less than 10% coverage2: In lav_data_full(data = data, group = group, cluster = cluster,  :  lavaan WARNING: due to missing values, some pairwise combinations have less than 10% coverage> summary(unconstrained.model.caudal.fit,fit.measures=TRUE, standardized=TRUE, rsquare=TRUE, ci=TRUE)lavaan 0.6-3 ended normally after 355 iterations  Optimization method                           NLMINB  Number of free parameters                         94  Number of equality constraints                    13  Number of observations per group           older                                            269  younger                                          336  Number of missing patterns per group       older                                             25  younger                                           24  Estimator                                         ML      Robust  Model Fit Test Statistic                     617.668     636.017  Degrees of freedom                               219         219  P-value (Chi-square)                           0.000       0.000  Scaling correction factor                                  0.971    for the Yuan-Bentler correction (Mplus variant)Chi-square for each group:  older                                        265.490     273.378  younger                                      352.177     362.640User model versus baseline model:  Comparative Fit Index (CFI)                       NA          NA  Tucker-Lewis Index (TLI)                          NA          NA  Robust Comparative Fit Index (CFI)                            NA  Robust Tucker-Lewis Index (TLI)                               NALoglikelihood and Information Criteria:  Loglikelihood user model (H0)               8912.214    8912.214  Scaling correction factor                                  1.115    for the MLR correction  Loglikelihood unrestricted model (H1)       9221.048    9221.048  Scaling correction factor                                  1.080    for the MLR correction  Number of free parameters                         81          81  Akaike (AIC)                              -17662.428  -17662.428  Bayesian (BIC)                            -17305.605  -17305.605  Sample-size adjusted Bayesian (BIC)       -17562.760  -17562.760Root Mean Square Error of Approximation:  RMSEA                                          0.078       0.079  90 Percent Confidence Interval          0.070  0.085       0.072  0.087  P-value RMSEA <= 0.05                          0.000       0.000  Robust RMSEA                                               0.078  90 Percent Confidence Interval                             0.071  0.085Standardized Root Mean Square Residual:  SRMR                                           0.117       0.117Parameter Estimates:  Information                                 Observed  Observed information based on                Hessian  Standard Errors                   Robust.huber.whiteGroup 1 [older]:Latent Variables:                   Estimate  Std.Err  z-value  P(>|z|) ci.lower ci.upper   Std.lv  Std.all  factor =~                                                                                   EMoneg            1.000                               1.000    1.000    0.128    0.743    EMvneg  (.p2.)    1.255    0.134    9.394    0.000    0.993    1.517    0.161    0.788    EMpneg  (.p3.)    0.082    0.072    1.142    0.253   -0.059    0.224    0.011    0.089    ERneg   (.p4.)    0.455    0.192    2.366    0.018    0.078    0.832    0.058    0.263    ERnegrc (.p5.)    0.020    0.020    1.000    0.317   -0.020    0.060    0.003    0.130    SSRTb   (.p6.)    0.034    0.068    0.506    0.613   -0.099    0.168    0.004    0.091    Htlts_T (.p7.)   -0.472    0.101   -4.691    0.000   -0.669   -0.275   -0.061   -0.326    PSQI_sc (.p8.)   -0.056    0.025   -2.221    0.026   -0.106   -0.007   -0.007   -0.186    edctn_g (.p9.)    0.160    0.032    5.061    0.000    0.098    0.222    0.021    0.446    Occ_scr (.10.)   -0.233    0.118   -1.977    0.048   -0.463   -0.002   -0.030   -0.198    STW_ttl (.11.)    0.151    0.043    3.497    0.000    0.066    0.235    0.019    0.350    Cttll.t (.12.)    0.307    0.054    5.670    0.000    0.201    0.414    0.039    0.668    smmbV_N (.13.)   -0.038    0.060   -0.629    0.529   -0.156    0.080   -0.005   -0.043    Fcs_FAM (.14.)    0.114    0.034    3.318    0.001    0.047    0.181    0.015    0.253Regressions:                   Estimate  Std.Err  z-value  P(>|z|) ci.lower ci.upper   Std.lv  Std.all  factor ~                                                                                    Rmeanlw (LC__)    0.489    0.286    1.708    0.088   -0.072    1.050    3.810    0.112    age_yrs          -0.814    0.148   -5.492    0.000   -1.104   -0.523   -6.341   -0.515  Rmeanlow ~                                                                                  age_yrs          -0.025    0.021   -1.153    0.249   -0.066    0.017   -0.025   -0.068Intercepts:                   Estimate  Std.Err  z-value  P(>|z|) ci.lower ci.upper   Std.lv  Std.all   .EMoneg            1.197    0.112   10.681    0.000    0.978    1.417    1.197    6.932   .EMvneg            1.059    0.148    7.145    0.000    0.768    1.349    1.059    5.182   .EMpneg            0.178    0.043    4.111    0.000    0.093    0.263    0.178    1.491   .ERneg             0.699    0.129    5.425    0.000    0.447    0.952    0.699    3.155   .ERnegreac         0.030    0.013    2.316    0.021    0.005    0.055    0.030    1.480   .SSRTb             0.199    0.035    5.673    0.000    0.130    0.268    0.199    4.120   .Hoteltask_Time    0.094    0.069    1.357    0.175   -0.042    0.230    0.094    0.506   .PSQI_score        0.027    0.012    2.292    0.022    0.004    0.050    0.027    0.695   .education_age     0.283    0.016   17.475    0.000    0.251    0.315    0.283    6.148   .Occ_score         0.095    0.053    1.784    0.074   -0.009    0.199    0.095    0.629   .STW_total         0.626    0.017   37.124    0.000    0.593    0.659    0.626   11.343   .Cattell.ttlscr    0.441    0.043   10.225    0.000    0.357    0.526    0.441    7.471   .semmbVsmnmb_pN    0.174    0.032    5.439    0.000    0.111    0.236    0.174    1.543   .Faces_FAMnam      0.270    0.024   11.033    0.000    0.222    0.318    0.270    4.672   .Rmeanlow          0.107    0.015    7.110    0.000    0.077    0.136    0.107    3.639   .factor            0.000                               0.000    0.000    0.000    0.000Variances:                   Estimate  Std.Err  z-value  P(>|z|) ci.lower ci.upper   Std.lv  Std.all   .EMoneg            0.013    0.002    5.623    0.000    0.009    0.018    0.013    0.448   .EMvneg            0.016    0.003    5.594    0.000    0.010    0.021    0.016    0.379   .EMpneg            0.014    0.002    9.144    0.000    0.011    0.017    0.014    0.992   .ERneg             0.046    0.010    4.549    0.000    0.026    0.065    0.046    0.931   .ERnegreac         0.000    0.000    6.859    0.000    0.000    0.001    0.000    0.983   .SSRTb             0.002    0.000    5.942    0.000    0.002    0.003    0.002    0.992   .Hoteltask_Time    0.031    0.003    9.490    0.000    0.025    0.037    0.031    0.894   .PSQI_score        0.001    0.000    8.808    0.000    0.001    0.002    0.001    0.966   .education_age     0.002    0.000    4.550    0.000    0.001    0.002    0.002    0.801   .Occ_score         0.022    0.003    7.075    0.000    0.016    0.028    0.022    0.961   .STW_total         0.003    0.000    5.927    0.000    0.002    0.004    0.003    0.877   .Cattell.ttlscr    0.002    0.000    7.145    0.000    0.001    0.002    0.002    0.554   .semmbVsmnmb_pN    0.013    0.002    8.023    0.000    0.010    0.016    0.013    0.998   .Faces_FAMnam      0.003    0.000   11.654    0.000    0.003    0.004    0.003    0.936   .Rmeanlow          0.001    0.000   10.848    0.000    0.001    0.001    0.001    0.995   .factor            0.012    0.003    3.776    0.000    0.006    0.018    0.715    0.715R-Square:                   Estimate    EMoneg            0.552    EMvneg            0.621    EMpneg            0.008    ERneg             0.069    ERnegreac         0.017    SSRTb             0.008    Hoteltask_Time    0.106    PSQI_score        0.034    education_age     0.199    Occ_score         0.039    STW_total         0.123    Cattell.ttlscr    0.446    semmbVsmnmb_pN    0.002    Faces_FAMnam      0.064    Rmeanlow          0.005    factor            0.285Group 2 [younger]:Latent Variables:                   Estimate  Std.Err  z-value  P(>|z|) ci.lower ci.upper   Std.lv  Std.all  factor =~                                                                                   EMoneg            1.000                               1.000    1.000    0.079    0.662    EMvneg  (.p2.)    1.255    0.134    9.394    0.000    0.993    1.517    0.099    0.633    EMpneg  (.p3.)    0.082    0.072    1.142    0.253   -0.059    0.224    0.006    0.064    ERneg   (.p4.)    0.455    0.192    2.366    0.018    0.078    0.832    0.036    0.206    ERnegrc (.p5.)    0.020    0.020    1.000    0.317   -0.020    0.060    0.002    0.091    SSRTb   (.p6.)    0.034    0.068    0.506    0.613   -0.099    0.168    0.003    0.065    Htlts_T (.p7.)   -0.472    0.101   -4.691    0.000   -0.669   -0.275   -0.037   -0.246    PSQI_sc (.p8.)   -0.056    0.025   -2.221    0.026   -0.106   -0.007   -0.004   -0.122    edctn_g (.p9.)    0.160    0.032    5.061    0.000    0.098    0.222    0.013    0.389    Occ_scr (.10.)   -0.233    0.118   -1.977    0.048   -0.463   -0.002   -0.018   -0.112    STW_ttl (.11.)    0.151    0.043    3.497    0.000    0.066    0.235    0.012    0.230    Cttll.t (.12.)    0.307    0.054    5.670    0.000    0.201    0.414    0.024    0.533    smmbV_N (.13.)   -0.038    0.060   -0.629    0.529   -0.156    0.080   -0.003   -0.029    Fcs_FAM (.14.)    0.114    0.034    3.318    0.001    0.047    0.181    0.009    0.194Regressions:                   Estimate  Std.Err  z-value  P(>|z|) ci.lower ci.upper   Std.lv  Std.all  factor ~                                                                                    Rmeanlw (LC__)   -0.338    0.261   -1.296    0.195   -0.849    0.173   -4.300   -0.107    age_yrs          -0.187    0.084   -2.216    0.027   -0.353   -0.022   -2.380   -0.246  Rmeanlow ~                                                                                  age_yrs           0.106    0.012    8.556    0.000    0.081    0.130    0.106    0.439Intercepts:                   Estimate  Std.Err  z-value  P(>|z|) ci.lower ci.upper   Std.lv  Std.all   .EMoneg            0.940    0.029   31.961    0.000    0.882    0.998    0.940    7.920   .EMvneg            0.777    0.041   19.167    0.000    0.698    0.857    0.777    4.989   .EMpneg            0.181    0.012   15.572    0.000    0.158    0.203    0.181    1.777   .ERneg             0.587    0.025   23.429    0.000    0.537    0.636    0.587    3.379   .ERnegreac         0.033    0.003   13.078    0.000    0.028    0.038    0.033    1.864   .SSRTb             0.171    0.008   20.489    0.000    0.154    0.187    0.171    4.132   .Hoteltask_Time    0.216    0.017   12.918    0.000    0.183    0.249    0.216    1.430   .PSQI_score        0.046    0.003   15.487    0.000    0.040    0.052    0.046    1.264   .education_age     0.230    0.005   49.146    0.000    0.221    0.239    0.230    7.127   .Occ_score         0.186    0.012   14.946    0.000    0.161    0.210    0.186    1.143   .STW_total         0.546    0.005  120.753    0.000    0.537    0.554    0.546   10.588   .Cattell.ttlscr    0.388    0.010   37.779    0.000    0.368    0.408    0.388    8.571   .semmbVsmnmb_pN    0.176    0.008   22.263    0.000    0.161    0.192    0.176    1.732   .Faces_FAMnam      0.270    0.005   58.725    0.000    0.261    0.279    0.270    5.873   .Rmeanlow          0.033    0.005    6.825    0.000    0.024    0.043    0.033    1.332   .factor            0.000                               0.000    0.000    0.000    0.000Variances:                   Estimate  Std.Err  z-value  P(>|z|) ci.lower ci.upper   Std.lv  Std.all   .EMoneg            0.008    0.002    4.756    0.000    0.005    0.011    0.008    0.562   .EMvneg            0.015    0.003    4.905    0.000    0.009    0.020    0.015    0.599   .EMpneg            0.010    0.001   10.398    0.000    0.008    0.012    0.010    0.996   .ERneg             0.029    0.003    9.125    0.000    0.023    0.035    0.029    0.958   .ERnegreac         0.000    0.000    8.894    0.000    0.000    0.000    0.000    0.992   .SSRTb             0.002    0.000    7.182    0.000    0.001    0.002    0.002    0.996   .Hoteltask_Time    0.021    0.002   11.504    0.000    0.018    0.025    0.021    0.940   .PSQI_score        0.001    0.000    8.324    0.000    0.001    0.002    0.001    0.985   .education_age     0.001    0.000    8.623    0.000    0.001    0.001    0.001    0.848   .Occ_score         0.026    0.003    7.855    0.000    0.020    0.033    0.026    0.987   .STW_total         0.003    0.000    6.244    0.000    0.002    0.003    0.003    0.947   .Cattell.ttlscr    0.001    0.000    9.310    0.000    0.001    0.002    0.001    0.715   .semmbVsmnmb_pN    0.010    0.001   12.891    0.000    0.009    0.012    0.010    0.999   .Faces_FAMnam      0.002    0.000    8.304    0.000    0.002    0.003    0.002    0.962   .Rmeanlow          0.000    0.000   10.961    0.000    0.000    0.001    0.000    0.807   .factor            0.006    0.002    2.901    0.004    0.002    0.009    0.905    0.905R-Square:                   Estimate    EMoneg            0.438    EMvneg            0.401    EMpneg            0.004    ERneg             0.042    ERnegreac         0.008    SSRTb             0.004    Hoteltask_Time    0.060    PSQI_score        0.015    education_age     0.152    Occ_score         0.013    STW_total         0.053    Cattell.ttlscr    0.285    semmbVsmnmb_pN    0.001    Faces_FAMnam      0.038    Rmeanlow          0.193    factor            0.095*NON AGE-ADJUSTED MULTIGROUP UNIDIMENSIONAL MODEL - CAUDAL LC> unconstrained.caudal.noage<-'+ factor=~ EMoneg+EMvneg +EMpneg+ERneg + ERnegreac +SSRTb+ Hoteltask_Time+ PSQI_score+education_age+Occ_score+STW_total+Cattell.totalscore+semambVsemunamb_pNo + Faces_FAMnam + factor ~ c(LC_effect_young,LC_effect_old)*Rmeanlow'> unconstrained.caudal.noage.fit<- cfa(unconstrained.caudal.noage, data=ourdata1,  group="agecat",group.equal = c("loadings"),missing = "ML", estimator='mlr')Warning messages:1: In lav_data_full(data = data, group = group, cluster = cluster,  :  lavaan WARNING: due to missing values, some pairwise combinations have less than 10% coverage2: In lav_data_full(data = data, group = group, cluster = cluster,  :  lavaan WARNING: due to missing values, some pairwise combinations have less than 10% coverage> summary(unconstrained.caudal.noage.fit,fit.measures=TRUE, standardized=TRUE, rsquare=TRUE, ci=TRUE)lavaan 0.6-3 ended normally after 320 iterations  Optimization method                           NLMINB  Number of free parameters                         86  Number of equality constraints                    13  Number of observations per group           older                                            269  younger                                          336  Number of missing patterns per group       older                                             25  younger                                           24  Estimator                                         ML      Robust  Model Fit Test Statistic                     383.436     395.605  Degrees of freedom                               193         193  P-value (Chi-square)                           0.000       0.000  Scaling correction factor                                  0.969    for the Yuan-Bentler correction (Mplus variant)Chi-square for each group:  older                                        156.576     161.545  younger                                      226.860     234.060User model versus baseline model:  Comparative Fit Index (CFI)                       NA          NA  Tucker-Lewis Index (TLI)                          NA          NA  Robust Comparative Fit Index (CFI)                            NA  Robust Tucker-Lewis Index (TLI)                               NALoglikelihood and Information Criteria:  Loglikelihood user model (H0)               7518.655    7518.655  Scaling correction factor                                  1.125    for the MLR correction  Loglikelihood unrestricted model (H1)       7710.373    7710.373  Scaling correction factor                                  1.092    for the MLR correction  Number of free parameters                         73          73  Akaike (AIC)                              -14891.311  -14891.311  Bayesian (BIC)                            -14569.729  -14569.729  Sample-size adjusted Bayesian (BIC)       -14801.486  -14801.486Root Mean Square Error of Approximation:  RMSEA                                          0.057       0.059  90 Percent Confidence Interval          0.049  0.065       0.050  0.067  P-value RMSEA <= 0.05                          0.080       0.041  Robust RMSEA                                               0.058  90 Percent Confidence Interval                             0.050  0.066Standardized Root Mean Square Residual:  SRMR                                           0.115       0.115Parameter Estimates:  Information                                 Observed  Observed information based on                Hessian  Standard Errors                   Robust.huber.whiteGroup 1 [older]:Latent Variables:                   Estimate  Std.Err  z-value  P(>|z|) ci.lower ci.upper   Std.lv  Std.all  factor =~                                                                                   EMoneg            1.000                               1.000    1.000    0.126    0.740    EMvneg  (.p2.)    1.226    0.128    9.548    0.000    0.974    1.478    0.155    0.767    EMpneg  (.p3.)    0.067    0.075    0.887    0.375   -0.081    0.214    0.008    0.070    ERneg   (.p4.)    0.275    0.213    1.292    0.196   -0.142    0.692    0.035    0.155    ERnegrc (.p5.)   -0.009    0.019   -0.453    0.651   -0.046    0.029   -0.001   -0.053    SSRTb   (.p6.)    0.076    0.073    1.045    0.296   -0.067    0.219    0.010    0.195    Htlts_T (.p7.)   -0.455    0.103   -4.411    0.000   -0.657   -0.253   -0.057   -0.308    PSQI_sc (.p8.)   -0.066    0.026   -2.505    0.012   -0.117   -0.014   -0.008   -0.213    edctn_g (.p9.)    0.185    0.034    5.424    0.000    0.118    0.252    0.023    0.514    Occ_scr (.10.)   -0.354    0.118   -3.004    0.003   -0.585   -0.123   -0.045   -0.296    STW_ttl (.11.)    0.215    0.040    5.343    0.000    0.136    0.294    0.027    0.494    Cttll.t (.12.)    0.256    0.049    5.248    0.000    0.160    0.351    0.032    0.549    smmbV_N (.13.)   -0.058    0.063   -0.910    0.363   -0.182    0.066   -0.007   -0.065    Fcs_FAM (.14.)    0.084    0.029    2.870    0.004    0.027    0.141    0.011    0.180Regressions:                   Estimate  Std.Err  z-value  P(>|z|) ci.lower ci.upper   Std.lv  Std.all  factor ~                                                                                    Rmeanlw (LC__)    0.688    0.313    2.200    0.028    0.075    1.301    5.454    0.160Intercepts:                   Estimate  Std.Err  z-value  P(>|z|) ci.lower ci.upper   Std.lv  Std.all   .EMoneg            0.596    0.033   18.136    0.000    0.532    0.661    0.596    3.500   .EMvneg            0.306    0.036    8.607    0.000    0.237    0.376    0.306    1.520   .EMpneg            0.129    0.011   11.568    0.000    0.108    0.151    0.129    1.084   .ERneg             0.437    0.025   17.297    0.000    0.387    0.486    0.437    1.949   .ERnegreac         0.019    0.002    9.501    0.000    0.015    0.023    0.019    0.943   .SSRTb             0.175    0.009   18.580    0.000    0.156    0.193    0.175    3.557   .Hoteltask_Time    0.376    0.017   22.566    0.000    0.344    0.409    0.376    2.018   .PSQI_score        0.061    0.004   15.965    0.000    0.054    0.069    0.061    1.572   .education_age     0.185    0.006   31.804    0.000    0.174    0.197    0.185    4.076   .Occ_score         0.242    0.017   14.375    0.000    0.209    0.275    0.242    1.605   .STW_total         0.532    0.008   69.819    0.000    0.517    0.547    0.532    9.684   .Cattell.ttlscr    0.260    0.008   31.087    0.000    0.243    0.276    0.260    4.419   .semmbVsmnmb_pN    0.198    0.008   23.767    0.000    0.181    0.214    0.198    1.755   .Faces_FAMnam      0.203    0.005   44.656    0.000    0.194    0.212    0.203    3.463   .factor            0.000                               0.000    0.000    0.000    0.000Variances:                   Estimate  Std.Err  z-value  P(>|z|) ci.lower ci.upper   Std.lv  Std.all   .EMoneg            0.013    0.003    4.867    0.000    0.008    0.018    0.013    0.452   .EMvneg            0.017    0.003    5.330    0.000    0.011    0.023    0.017    0.411   .EMpneg            0.014    0.002    9.159    0.000    0.011    0.017    0.014    0.995   .ERneg             0.049    0.010    4.840    0.000    0.029    0.069    0.049    0.976   .ERnegreac         0.000    0.000    6.358    0.000    0.000    0.001    0.000    0.997   .SSRTb             0.002    0.000    5.588    0.000    0.002    0.003    0.002    0.962   .Hoteltask_Time    0.031    0.003    9.540    0.000    0.025    0.038    0.031    0.905   .PSQI_score        0.001    0.000    8.855    0.000    0.001    0.002    0.001    0.955   .education_age     0.002    0.000    4.231    0.000    0.001    0.002    0.002    0.736   .Occ_score         0.021    0.003    6.598    0.000    0.015    0.027    0.021    0.913   .STW_total         0.002    0.000    5.262    0.000    0.001    0.003    0.002    0.756   .Cattell.ttlscr    0.002    0.000    8.032    0.000    0.002    0.003    0.002    0.699   .semmbVsmnmb_pN    0.013    0.002    7.999    0.000    0.010    0.016    0.013    0.996   .Faces_FAMnam      0.003    0.000   13.114    0.000    0.003    0.004    0.003    0.968   .factor            0.015    0.004    4.026    0.000    0.008    0.023    0.974    0.974R-Square:                   Estimate    EMoneg            0.548    EMvneg            0.589    EMpneg            0.005    ERneg             0.024    ERnegreac         0.003    SSRTb             0.038    Hoteltask_Time    0.095    PSQI_score        0.045    education_age     0.264    Occ_score         0.087    STW_total         0.244    Cattell.ttlscr    0.301    semmbVsmnmb_pN    0.004    Faces_FAMnam      0.032    factor            0.026Group 2 [younger]:Latent Variables:                   Estimate  Std.Err  z-value  P(>|z|) ci.lower ci.upper   Std.lv  Std.all  factor =~                                                                                   EMoneg            1.000                               1.000    1.000    0.084    0.689    EMvneg  (.p2.)    1.226    0.128    9.548    0.000    0.974    1.478    0.103    0.645    EMpneg  (.p3.)    0.067    0.075    0.887    0.375   -0.081    0.214    0.006    0.055    ERneg   (.p4.)    0.275    0.213    1.292    0.196   -0.142    0.692    0.023    0.135    ERnegrc (.p5.)   -0.009    0.019   -0.453    0.651   -0.046    0.029   -0.001   -0.042    SSRTb   (.p6.)    0.076    0.073    1.045    0.296   -0.067    0.219    0.006    0.156    Htlts_T (.p7.)   -0.455    0.103   -4.411    0.000   -0.657   -0.253   -0.038   -0.255    PSQI_sc (.p8.)   -0.066    0.026   -2.505    0.012   -0.117   -0.014   -0.006   -0.154    edctn_g (.p9.)    0.185    0.034    5.424    0.000    0.118    0.252    0.016    0.480    Occ_scr (.10.)   -0.354    0.118   -3.004    0.003   -0.585   -0.123   -0.030   -0.183    STW_ttl (.11.)    0.215    0.040    5.343    0.000    0.136    0.294    0.018    0.350    Cttll.t (.12.)    0.256    0.049    5.248    0.000    0.160    0.351    0.022    0.476    smmbV_N (.13.)   -0.058    0.063   -0.910    0.363   -0.182    0.066   -0.005   -0.048    Fcs_FAM (.14.)    0.084    0.029    2.870    0.004    0.027    0.141    0.007    0.156Regressions:                   Estimate  Std.Err  z-value  P(>|z|) ci.lower ci.upper   Std.lv  Std.all  factor ~                                                                                    Rmeanlw (LC__)   -0.636    0.243   -2.616    0.009   -1.112   -0.159   -7.536   -0.187Intercepts:                   Estimate  Std.Err  z-value  P(>|z|) ci.lower ci.upper   Std.lv  Std.all   .EMoneg            0.887    0.020   43.855    0.000    0.847    0.926    0.887    7.241   .EMvneg            0.709    0.027   26.261    0.000    0.656    0.762    0.709    4.419   .EMpneg            0.176    0.009   19.023    0.000    0.157    0.194    0.176    1.727   .ERneg             0.555    0.016   35.498    0.000    0.524    0.586    0.555    3.230   .ERnegreac         0.030    0.002   19.443    0.000    0.027    0.033    0.030    1.750   .SSRTb             0.171    0.006   27.086    0.000    0.158    0.183    0.171    4.164   .Hoteltask_Time    0.241    0.012   19.626    0.000    0.217    0.265    0.241    1.603   .PSQI_score        0.048    0.002   20.369    0.000    0.044    0.053    0.048    1.337   .education_age     0.223    0.004   60.630    0.000    0.216    0.230    0.223    6.836   .Occ_score         0.192    0.011   18.024    0.000    0.171    0.213    0.192    1.181   .STW_total         0.541    0.004  122.169    0.000    0.532    0.550    0.541   10.444   .Cattell.ttlscr    0.370    0.005   67.735    0.000    0.359    0.380    0.370    8.152   .semmbVsmnmb_pN    0.177    0.006   28.237    0.000    0.165    0.189    0.177    1.743   .Faces_FAMnam      0.262    0.003   85.195    0.000    0.256    0.268    0.262    5.798   .factor            0.000                               0.000    0.000    0.000    0.000Variances:                   Estimate  Std.Err  z-value  P(>|z|) ci.lower ci.upper   Std.lv  Std.all   .EMoneg            0.008    0.002    4.526    0.000    0.004    0.011    0.008    0.525   .EMvneg            0.015    0.003    4.951    0.000    0.009    0.021    0.015    0.584   .EMpneg            0.010    0.001   10.414    0.000    0.008    0.012    0.010    0.997   .ERneg             0.029    0.003    9.206    0.000    0.023    0.035    0.029    0.982   .ERnegreac         0.000    0.000    8.699    0.000    0.000    0.000    0.000    0.998   .SSRTb             0.002    0.000    7.010    0.000    0.001    0.002    0.002    0.976   .Hoteltask_Time    0.021    0.002   11.551    0.000    0.018    0.025    0.021    0.935   .PSQI_score        0.001    0.000    8.421    0.000    0.001    0.002    0.001    0.976   .education_age     0.001    0.000    8.131    0.000    0.001    0.001    0.001    0.770   .Occ_score         0.026    0.003    7.977    0.000    0.019    0.032    0.026    0.966   .STW_total         0.002    0.000    6.054    0.000    0.002    0.003    0.002    0.877   .Cattell.ttlscr    0.002    0.000   11.046    0.000    0.001    0.002    0.002    0.773   .semmbVsmnmb_pN    0.010    0.001   12.812    0.000    0.009    0.012    0.010    0.998   .Faces_FAMnam      0.002    0.000    8.399    0.000    0.002    0.002    0.002    0.976   .factor            0.007    0.002    3.154    0.002    0.003    0.011    0.965    0.965R-Square:                   Estimate    EMoneg            0.475    EMvneg            0.416    EMpneg            0.003    ERneg             0.018    ERnegreac         0.002    SSRTb             0.024    Hoteltask_Time    0.065    PSQI_score        0.024    education_age     0.230    Occ_score         0.034    STW_total         0.123    Cattell.ttlscr    0.227    semmbVsmnmb_pN    0.002    Faces_FAMnam      0.024    factor            0.035
