# Supplementary material for: Noradrenergic-dependent functions are associated with age-related locus coeruleus signal intensity differences
Source: Nat Commun. 2020 Apr 6;11:1712. doi: 10.1038/s41467-020-15410-w (PMC7136271; doi:10.1038/s41467-020-15410-w)
Supplement: Supplementary file 3 — Source Data [file 41467_2020_15410_MOESM3_ESM.zip › Source Data Table 3.rtf]

*UNIDIMENSIONAL AGE ADJUSTED MULTIGROUP MODEL  (UNCONSTRAINED)> unconstrained.model.uni<-'+ factor=~ EMoneg+EMvneg +EMpneg+ERneg + ERnegreac +SSRTb+ Hoteltask_Time+ PSQI_score+education_age+Occ_score+STW_total+Cattell.totalscore+semambVsemunamb_pNo + Faces_FAMnam + factor ~ c(LC_effect_young,LC_effect_old)*meRLC+ factor~age_years+ meRLC~age_years'> unconstrained.model.uni.fit<- cfa(unconstrained.model.uni, data=ourdata1,  group="agecat",group.equal = c("loadings"),missing = "ML", estimator='mlr')Warning messages:1: In lav_data_full(data = data, group = group, cluster = cluster,  :  lavaan WARNING: due to missing values, some pairwise combinations have less than 10% coverage2: In lav_data_full(data = data, group = group, cluster = cluster,  :  lavaan WARNING: due to missing values, some pairwise combinations have less than 10% coverage> summary(unconstrained.model.uni.fit,fit.measures=TRUE, standardized=TRUE, rsquare=TRUE, ci=TRUE)lavaan 0.6-3 ended normally after 369 iterations  Optimization method                           NLMINB  Number of free parameters                         94  Number of equality constraints                    13  Number of observations per group           older                                            269  younger                                          336  Number of missing patterns per group       older                                             25  younger                                           24  Estimator                                         ML      Robust  Model Fit Test Statistic                     619.820     635.984  Degrees of freedom                               219         219  P-value (Chi-square)                           0.000       0.000  Scaling correction factor                                  0.975    for the Yuan-Bentler correction (Mplus variant)Chi-square for each group:  older                                        265.642     272.570  younger                                      354.178     363.414User model versus baseline model:  Comparative Fit Index (CFI)                       NA          NA  Tucker-Lewis Index (TLI)                          NA          NA  Robust Comparative Fit Index (CFI)                            NA  Robust Tucker-Lewis Index (TLI)                               NALoglikelihood and Information Criteria:  Loglikelihood user model (H0)               8915.016    8915.016  Scaling correction factor                                  1.119    for the MLR correction  Loglikelihood unrestricted model (H1)       9224.927    9224.927  Scaling correction factor                                  1.084    for the MLR correction  Number of free parameters                         81          81  Akaike (AIC)                              -17668.033  -17668.033  Bayesian (BIC)                            -17311.209  -17311.209  Sample-size adjusted Bayesian (BIC)       -17568.364  -17568.364Root Mean Square Error of Approximation:  RMSEA                                          0.078       0.079  90 Percent Confidence Interval          0.071  0.085       0.072  0.087  P-value RMSEA <= 0.05                          0.000       0.000  Robust RMSEA                                               0.078  90 Percent Confidence Interval                             0.071  0.085Standardized Root Mean Square Residual:  SRMR                                           0.117       0.117Parameter Estimates:  Information                                 Observed  Observed information based on                Hessian  Standard Errors                   Robust.huber.whiteGroup 1 [older]:Latent Variables:                   Estimate  Std.Err  z-value  P(>|z|) ci.lower ci.upper   Std.lv  Std.all  factor =~                                                                                   EMoneg            1.000                               1.000    1.000    0.129    0.744    EMvneg  (.p2.)    1.255    0.133    9.422    0.000    0.994    1.516    0.161    0.789    EMpneg  (.p3.)    0.082    0.072    1.136    0.256   -0.059    0.223    0.010    0.088    ERneg   (.p4.)    0.462    0.191    2.417    0.016    0.087    0.836    0.059    0.268    ERnegrc (.p5.)    0.020    0.020    0.993    0.321   -0.020    0.060    0.003    0.129    SSRTb   (.p6.)    0.036    0.069    0.522    0.602   -0.099    0.172    0.005    0.096    Htlts_T (.p7.)   -0.466    0.099   -4.702    0.000   -0.661   -0.272   -0.060   -0.323    PSQI_sc (.p8.)   -0.056    0.025   -2.226    0.026   -0.106   -0.007   -0.007   -0.186    edctn_g (.p9.)    0.160    0.031    5.082    0.000    0.098    0.221    0.021    0.446    Occ_scr (.10.)   -0.233    0.118   -1.978    0.048   -0.464   -0.002   -0.030   -0.198    STW_ttl (.11.)    0.151    0.043    3.515    0.000    0.067    0.235    0.019    0.351    Cttll.t (.12.)    0.307    0.054    5.725    0.000    0.202    0.412    0.039    0.668    smmbV_N (.13.)   -0.036    0.060   -0.602    0.547   -0.154    0.081   -0.005   -0.041    Fcs_FAM (.14.)    0.112    0.034    3.295    0.001    0.045    0.179    0.014    0.249Regressions:                   Estimate  Std.Err  z-value  P(>|z|) ci.lower ci.upper   Std.lv  Std.all  factor ~                                                                                    meRLC   (LC__)    0.678    0.312    2.173    0.030    0.067    1.290    5.277    0.156    age_yrs          -0.804    0.148   -5.429    0.000   -1.094   -0.513   -6.253   -0.508  meRLC ~                                                                                     age_yrs          -0.032    0.021   -1.516    0.129   -0.073    0.009   -0.032   -0.087Intercepts:                   Estimate  Std.Err  z-value  P(>|z|) ci.lower ci.upper   Std.lv  Std.all   .EMoneg            1.166    0.115   10.145    0.000    0.941    1.391    1.166    6.748   .EMvneg            1.019    0.151    6.745    0.000    0.723    1.315    1.019    4.988   .EMpneg            0.175    0.041    4.276    0.000    0.095    0.255    0.175    1.467   .ERneg             0.689    0.125    5.513    0.000    0.444    0.934    0.689    3.107   .ERnegreac         0.029    0.012    2.370    0.018    0.005    0.053    0.029    1.441   .SSRTb             0.199    0.033    5.940    0.000    0.133    0.265    0.199    4.112   .Hoteltask_Time    0.111    0.068    1.627    0.104   -0.023    0.245    0.111    0.599   .PSQI_score        0.029    0.011    2.598    0.009    0.007    0.051    0.029    0.741   .education_age     0.278    0.016   16.851    0.000    0.246    0.310    0.278    6.040   .Occ_score         0.102    0.050    2.042    0.041    0.004    0.200    0.102    0.676   .STW_total         0.622    0.016   38.254    0.000    0.590    0.654    0.622   11.264   .Cattell.ttlscr    0.431    0.043    9.917    0.000    0.346    0.516    0.431    7.305   .semmbVsmnmb_pN    0.176    0.030    5.841    0.000    0.117    0.235    0.176    1.562   .Faces_FAMnam      0.265    0.024   11.131    0.000    0.219    0.312    0.265    4.595   .meRLC             0.122    0.015    8.194    0.000    0.093    0.151    0.122    4.120   .factor            0.000                               0.000    0.000    0.000    0.000Variances:                   Estimate  Std.Err  z-value  P(>|z|) ci.lower ci.upper   Std.lv  Std.all   .EMoneg            0.013    0.002    5.662    0.000    0.009    0.018    0.013    0.447   .EMvneg            0.016    0.003    5.623    0.000    0.010    0.021    0.016    0.378   .EMpneg            0.014    0.002    9.145    0.000    0.011    0.017    0.014    0.992   .ERneg             0.046    0.010    4.548    0.000    0.026    0.065    0.046    0.928   .ERnegreac         0.000    0.000    6.828    0.000    0.000    0.001    0.000    0.983   .SSRTb             0.002    0.000    5.933    0.000    0.002    0.003    0.002    0.991   .Hoteltask_Time    0.031    0.003    9.502    0.000    0.025    0.037    0.031    0.896   .PSQI_score        0.001    0.000    8.815    0.000    0.001    0.002    0.001    0.966   .education_age     0.002    0.000    4.565    0.000    0.001    0.002    0.002    0.801   .Occ_score         0.022    0.003    7.069    0.000    0.016    0.028    0.022    0.961   .STW_total         0.003    0.000    5.923    0.000    0.002    0.004    0.003    0.877   .Cattell.ttlscr    0.002    0.000    7.149    0.000    0.001    0.002    0.002    0.554   .semmbVsmnmb_pN    0.013    0.002    8.024    0.000    0.010    0.016    0.013    0.998   .Faces_FAMnam      0.003    0.000   11.675    0.000    0.003    0.004    0.003    0.938   .meRLC             0.001    0.000   10.119    0.000    0.001    0.001    0.001    0.992   .factor            0.012    0.003    3.839    0.000    0.006    0.018    0.704    0.704R-Square:                   Estimate    EMoneg            0.553    EMvneg            0.622    EMpneg            0.008    ERneg             0.072    ERnegreac         0.017    SSRTb             0.009    Hoteltask_Time    0.104    PSQI_score        0.034    education_age     0.199    Occ_score         0.039    STW_total         0.123    Cattell.ttlscr    0.446    semmbVsmnmb_pN    0.002    Faces_FAMnam      0.062    meRLC             0.008    factor            0.296Group 2 [younger]:Latent Variables:                   Estimate  Std.Err  z-value  P(>|z|) ci.lower ci.upper   Std.lv  Std.all  factor =~                                                                                   EMoneg            1.000                               1.000    1.000    0.079    0.663    EMvneg  (.p2.)    1.255    0.133    9.422    0.000    0.994    1.516    0.099    0.634    EMpneg  (.p3.)    0.082    0.072    1.136    0.256   -0.059    0.223    0.006    0.063    ERneg   (.p4.)    0.462    0.191    2.417    0.016    0.087    0.836    0.036    0.209    ERnegrc (.p5.)    0.020    0.020    0.993    0.321   -0.020    0.060    0.002    0.090    SSRTb   (.p6.)    0.036    0.069    0.522    0.602   -0.099    0.172    0.003    0.069    Htlts_T (.p7.)   -0.466    0.099   -4.702    0.000   -0.661   -0.272   -0.037   -0.243    PSQI_sc (.p8.)   -0.056    0.025   -2.226    0.026   -0.106   -0.007   -0.004   -0.122    edctn_g (.p9.)    0.160    0.031    5.082    0.000    0.098    0.221    0.013    0.389    Occ_scr (.10.)   -0.233    0.118   -1.978    0.048   -0.464   -0.002   -0.018   -0.113    STW_ttl (.11.)    0.151    0.043    3.515    0.000    0.067    0.235    0.012    0.230    Cttll.t (.12.)    0.307    0.054    5.725    0.000    0.202    0.412    0.024    0.532    smmbV_N (.13.)   -0.036    0.060   -0.602    0.547   -0.154    0.081   -0.003   -0.028    Fcs_FAM (.14.)    0.112    0.034    3.295    0.001    0.045    0.179    0.009    0.192Regressions:                   Estimate  Std.Err  z-value  P(>|z|) ci.lower ci.upper   Std.lv  Std.all  factor ~                                                                                    meRLC   (LC__)   -0.257    0.259   -0.995    0.320   -0.765    0.250   -3.277   -0.080    age_yrs          -0.197    0.084   -2.357    0.018   -0.361   -0.033   -2.509   -0.260  meRLC ~                                                                                     age_yrs           0.104    0.012    8.377    0.000    0.080    0.128    0.104    0.438Intercepts:                   Estimate  Std.Err  z-value  P(>|z|) ci.lower ci.upper   Std.lv  Std.all   .EMoneg            0.941    0.030   31.753    0.000    0.883    0.999    0.941    7.941   .EMvneg            0.778    0.041   19.080    0.000    0.698    0.858    0.778    5.005   .EMpneg            0.181    0.012   15.548    0.000    0.158    0.204    0.181    1.777   .ERneg             0.588    0.025   23.298    0.000    0.538    0.637    0.588    3.385   .ERnegreac         0.033    0.003   13.083    0.000    0.028    0.038    0.033    1.864   .SSRTb             0.171    0.009   20.112    0.000    0.154    0.188    0.171    4.139   .Hoteltask_Time    0.216    0.017   12.965    0.000    0.183    0.248    0.216    1.430   .PSQI_score        0.046    0.003   15.397    0.000    0.040    0.052    0.046    1.263   .education_age     0.230    0.005   48.978    0.000    0.221    0.239    0.230    7.129   .Occ_score         0.186    0.013   14.704    0.000    0.161    0.210    0.186    1.141   .STW_total         0.546    0.005  118.049    0.000    0.537    0.555    0.546   10.587   .Cattell.ttlscr    0.388    0.010   38.070    0.000    0.368    0.408    0.388    8.576   .semmbVsmnmb_pN    0.176    0.008   22.214    0.000    0.161    0.192    0.176    1.733   .Faces_FAMnam      0.270    0.005   58.182    0.000    0.261    0.279    0.270    5.874   .meRLC             0.046    0.005    9.447    0.000    0.036    0.055    0.046    1.860   .factor            0.000                               0.000    0.000    0.000    0.000Variances:                   Estimate  Std.Err  z-value  P(>|z|) ci.lower ci.upper   Std.lv  Std.all   .EMoneg            0.008    0.002    4.791    0.000    0.005    0.011    0.008    0.560   .EMvneg            0.014    0.003    4.941    0.000    0.009    0.020    0.014    0.598   .EMpneg            0.010    0.001   10.401    0.000    0.008    0.012    0.010    0.996   .ERneg             0.029    0.003    9.114    0.000    0.023    0.035    0.029    0.956   .ERnegreac         0.000    0.000    8.896    0.000    0.000    0.000    0.000    0.992   .SSRTb             0.002    0.000    7.176    0.000    0.001    0.002    0.002    0.995   .Hoteltask_Time    0.021    0.002   11.516    0.000    0.018    0.025    0.021    0.941   .PSQI_score        0.001    0.000    8.312    0.000    0.001    0.002    0.001    0.985   .education_age     0.001    0.000    8.644    0.000    0.001    0.001    0.001    0.849   .Occ_score         0.026    0.003    7.853    0.000    0.020    0.033    0.026    0.987   .STW_total         0.003    0.000    6.235    0.000    0.002    0.003    0.003    0.947   .Cattell.ttlscr    0.001    0.000    9.325    0.000    0.001    0.002    0.001    0.717   .semmbVsmnmb_pN    0.010    0.001   12.896    0.000    0.009    0.012    0.010    0.999   .Faces_FAMnam      0.002    0.000    8.301    0.000    0.002    0.003    0.002    0.963   .meRLC             0.000    0.000   10.401    0.000    0.000    0.001    0.000    0.809   .factor            0.006    0.002    2.932    0.003    0.002    0.009    0.908    0.908R-Square:                   Estimate    EMoneg            0.440    EMvneg            0.402    EMpneg            0.004    ERneg             0.044    ERnegreac         0.008    SSRTb             0.005    Hoteltask_Time    0.059    PSQI_score        0.015    education_age     0.151    Occ_score         0.013    STW_total         0.053    Cattell.ttlscr    0.283    semmbVsmnmb_pN    0.001    Faces_FAMnam      0.037    meRLC             0.191    factor            0.092*COMPARE REGRESSION PATHS BETWEEN LC AND FACTOR IN ABOVE MODEL TO A CONSTRAINED MODEL> constrained.model.uni<-'+ factor=~ EMoneg+EMvneg +EMpneg+ERneg + ERnegreac +SSRTb+ Hoteltask_Time+ PSQI_score+education_age+Occ_score+STW_total+Cattell.totalscore+semambVsemunamb_pNo + Faces_FAMnam + factor ~ c(LC_effect,LC_effect)*meRLC+ factor~age_years+ meRLC~age_years'> constrained.model.uni.fit<- cfa(constrained.model.uni, data=ourdata1,  group="agecat",group.equal = c("loadings"),missing = "ML", estimator='mlr')Warning messages:1: In lav_data_full(data = data, group = group, cluster = cluster,  :  lavaan WARNING: due to missing values, some pairwise combinations have less than 10% coverage2: In lav_data_full(data = data, group = group, cluster = cluster,  :  lavaan WARNING: due to missing values, some pairwise combinations have less than 10% coverage> anova(constrained.model.uni.fit,unconstrained.model.uni.fit)Scaled Chi Square Difference Test (method = "satorra.bentler.2001")                             Df    AIC    BIC  Chisq Chisq diff Df diff Pr(>Chisq)  unconstrained.model.uni.fit 219 -17668 -17311 619.82                                constrained.model.uni.fit   220 -17664 -17312 625.82     5.6894       1    0.01707 *---Signif. codes:  0 ‘***’ 0.001 ‘**’ 0.01 ‘*’ 0.05 ‘.’ 0.1 ‘ ’ 1
